# Current status of the cryopreservation of embryogenic material of woody species

**DOI:** 10.3389/fpls.2023.1337152

**Published:** 2024-01-17

**Authors:** Daniel Ballesteros, María Teresa Martínez, Carolina Sánchez-Romero, Itziar Aurora Montalbán, Ester Sales, Paloma Moncaleán, Isabel Arrillaga, Elena Corredoira

**Affiliations:** ^1^ Departamento de Botánica y Geología, Facultad de Farmacia, Universitat de València, Burjassot, Valencia, Spain; ^2^ Royal Botanic Gardens, Kew, Wakehurst Place, Haywards Heath, United Kingdom; ^3^ Misión Biológica de Galicia (MBG-CSIC), Sede Santiago de Compostela, Santiago de Compostela, Spain; ^4^ Dpto. de Botánica y Fisiología Vegetal, Universidad de Málaga, Málaga, Spain; ^5^ Depto. Ciencias Forestales, Neiker-BRTA, Vitoria, Spain; ^6^ Dpto. Ciencias Agrarias y del Medio natural, Instituto Universitario de Investigación en Ciencias Ambientales (IUCA), Universidad de Zaragoza, Escuela Politécnica Superior, Huesca, Spain; ^7^ Institut Biotec/Med, Dpto Biología Vegetal, Facultad de Farmacia, Universitat de València, Burjassot, Valencia, Spain

**Keywords:** conifers, deciduous forest species, fruit species, genetic stability, liquid nitrogen, slow cooling, somatic embryos, vitrification

## Abstract

Cryopreservation, or the storage at liquid nitrogen temperatures (-196°C), of embryogenic cells or somatic embryos allows their long-term conservation without loss of their embryogenic capacity. During the last decade, protocols for cryopreservation of embryogenic material of woody species have been increasing in number and importance. However, despite the large experimental evidence proved in thousands of embryogenic lines, the application for the large-scale conservation of embryogenic material in cryobanks is still limited. Cryopreservation facilitates the management of embryogenic lines, reducing costs and time spent on their maintenance, thus limiting the risk of the appearance of somaclonal variation or contamination. Somatic embryogenesis in combination with cryopreservation is especially useful to preserve the juvenility of lines while the corresponding clones are being field-tested. Hence, when tree performance has been evaluated, selected varieties can be propagated from the cryostock. The traditional method of slow cooling or techniques based on vitrification are mostly applied procedures. For example, slow cooling methods are widely applied to conserve embryogenic lines of conifers. Desiccation based procedures, although simpler, have been applied in a smaller number of species. Genetic stability of the cryopreserved material is supported by multiloci PCR-derived markers in most of the assayed species, whereas DNA methylation status assays showed that cryopreservation might induce some changes that were also observed after prolonged subculture of the embryogenic lines. This article reviews the cryopreservation of embryogenic cultures in conifers, fruit species, deciduous forest species and palms, including a description of the different cryopreservation procedures and the analysis of their genetic stability after storage in liquid nitrogen.

## 1 Introduction

The ecological and economic value of woody species is unquestionable. They provide fruits, timber and other non-wood products. Moreover, forest ecosystems help stabilize the climate, protect the biodiversity of microbes, fauna and flora, and offer cultural and recreational services (Agriculture, Forestry and Fishery Statistics, 2019). Besides, trees and forests are keystones in mitigating climate change by absorbing the carbon dioxide emitted from human activities (Kay et al., 2019; Waring et al., 2020). Lastly, biomass from trees is increasingly used as a renewable energy source (Sahoo et al., 2022). However, the increasing needs on both timber and non-timber forest resources, deforestation, diseases, pests, global climate change and the low natural regeneration capacity of many woody species seriously threaten both agroforestry systems and forestry in general. Conservation of woody species is essential for preserving genetic biodiversity, for tree breeding and for maintaining valuable material for investigation (Corredoira et al., 2017a; Corredoira et al., 2017b).

The *ex situ* conservation of plant genetic resources is generally accomplished through the cold storage of dry seeds in germplasm banks (Walters and Pence, 2021). This is a relatively low-tech methodology for which international standards have been set up (e.g., FAO, 2014; CPC, Center for Plant Conservation, 2019). It is potentially effective for near 90% of all seeded plants (Wyse and Dickie, 2017) and used in over 1700 seed banks globally (Breman et al., 2021; Walters and Pence, 2021). However, not all the seeds survive the dry and cold conditions of seed banks. For example, a third of all tree species in the world are predicted to have desiccation and freezing sensitive seeds (i.e., recalcitrant seeds, Wyse et al., 2018), a proportion that increases to near 50% of non-pioneer evergreen rain forest trees (Tweddle et al., 2003). In addition, for some plant genetic resources (e.g., clonal crops or timber) it is important the preservation of specific genotypes, something that cannot be achieved through seed banking because of the heterozygosity of seeds (Engelmann, 2004). In such cases, the combined use of cryopreservation and micropropagation is required (Pence et al., 2020; Walters and Pence, 2021).

Cryopreservation is the preservation of cells, tissues, organs, or even full organisms such as seeds at very low temperatures. It is often achieved by the storage of these biological materials in liquid nitrogen (LN, -196°C) or in its vapor phase (<-160°C) (Benelli, 2021; Walters and Pence, 2021). Successful cryopreservation of plant explants aims to inhibit cell metabolism, stabilize cellular structures and limit any molecular motion and chemical activity by solidifying the aqueous cytoplasm without the formation of ice crystals (Benson, 2008a; Walters and Pence, 2021). This process is known as “vitrification” (aka formation of a glassy state) and is easily achieved in desiccation tolerant seeds and other explants that vitrify during natural drying at dispersal or during the drying imposed by the gene bank (Ballesteros et al., 2021; Walters and Pence, 2021). When plant explants do not tolerate sufficient drying to remove the fraction of water that can form intracellular lethal ice crystals upon cooling, cytoplasmic vitrification requires partial dehydration (Walters and Pence, 2021). This can be achieved through the induction of extracellular ice formation that favors intracellular water migration to the extracellular space, physical air drying of the explant, or (and) the addition of osmotic and cryoprotectant molecules that remove/replace water and induce a glassy state formation within the cytoplasm without massive cellular deformation (Benson, 2008a; Walters and Pence, 2021). These procedures are followed by relatively fast cooling at rates of a few hundred °C/sec (Walters and Pence, 2021). Specific approaches and molecules for cryoprotection in plant cryopreservation will be detailed in section 4.

Micropropagation (i.e., clonal multiplication *in vitro*) includes, as defined in Bornman (1993), “three vegetative propagation techniques: (1) axillary budding from explants containing pre-existing meristems; (2) caulogenesis or adventitious bud induction following induction of adventitious meristems; and (3) somatic embryogenesis (SE)”. SE is defined as the process by which plants can develop bipolar structures called somatic embryos from somatic cells (Corredoira et al., 2019). Nowadays, SE induction from immature zygotic embryos has become a routine procedure for some woody species, and in particular for some coniferous species (Pais, 2019). SE plays a critical role in clonal propagation; hence, it is the principal and most effective procedure for regenerating any type of cell or tissue that has been genetically transformed or cryopreserved (Corredoira and Costa, 2021). These three tools (i.e., SE, cryopreservation and genetic transformation) present enormous potential for the improvement of woody species, especially in the case of forest species. This is mostly because SE produces higher proliferation rates than any other micropropagation procedures, and also because only one alive embryogenic cell can regenerate a full somatic embryo (Corredoira et al., 2019). In addition, the high proliferation ability of embryogenic cultures also provides high quantities of somatic embryos to test how the material will be affected by the different cryopreservation procedures, which permits to develop solid protocols (Engelmann, 2000).

The implementation of cryopreservation techniques for the conservation of woody species’ *in vitro* cultures is relatively recent, with the first report published in the early 1990s (Sakai et al., 1990). Nonetheless, somatic embryos of diverse woody species have been successfully cryopreserved since then. Cryopreservation is particularly useful for the management of embryogenic cultures. Once an embryogenic line has been established, periodic subculture is necessary to maintain the embryogenic ability. This task is labor-intensive and there are also a risks of losing lines by contamination, technical failure, human mistakes, reduction of the embryogenic ability, or the appearance of somaclonal variation (Bradaï et al., 2023). The development and optimization of efficient cryopreservation procedures enable safe and long-term conservation of embryogenic cultures (Ozudogru and Lambardi, 2016). Cryopreservation of somatic embryos is also valuable for further biotechnological manipulations and for storage of biotechnological products such as genetically transformed or edited lines (Corredoira et al., 2017a). Moreover, cryopreservation provides an opportunity to create clone cryobanks of selected lines for commercial clonal forestry (Park et al., 2016). Cryopreservation coupled with SE is particularly useful to improve conifers. The combination of both tools is essential for the implementation of Multi-Varietal Forestry (MVF), which is described as the use of a range of genetically evaluated tree clones (i.e. plus trees) in commercial plantation forestry (Park et al., 2016). SE is induced in selected seeds derived from plus trees, and the embryogenic lines are cryopreserved until the progeny tests results are obtained (Nunes et al., 2017).

The present report reviews the current status of cryopreservation techniques applied to conserve somatic embryogenic tissues of the main woody species, with special attention the principal physical and technological aspects involved in their cryopreservation.

## 2 Selection of initial explant

**Figure 1:**
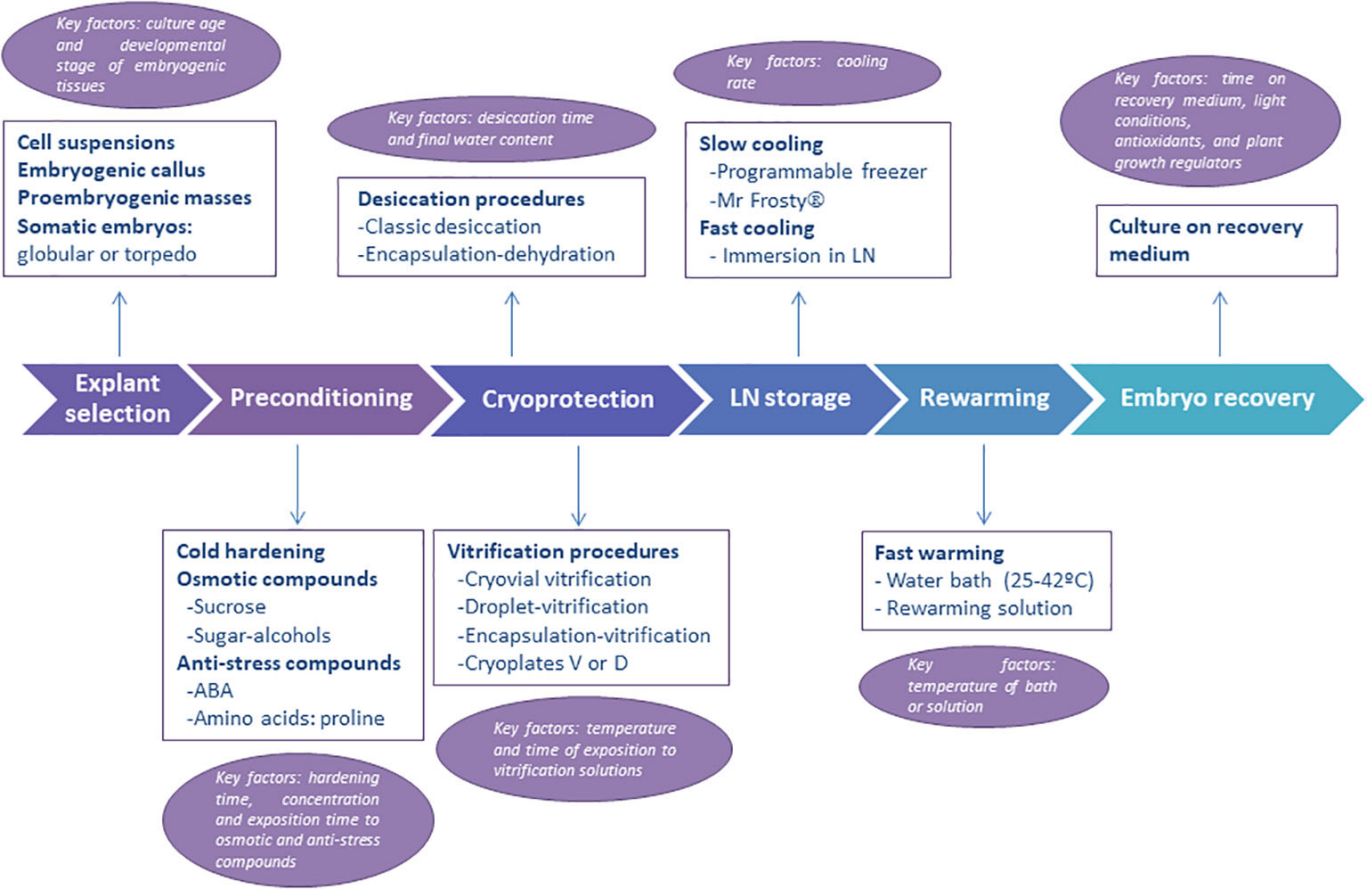
Schematic representation of the steps followed in the cryopresevation of embryogenic material. Information on explant types, preconditioning, cryoprotection, cooling and warming procedures are given. The main key factors to consider (i.e., optimize) at each step are also provided.

**Figure 2:**
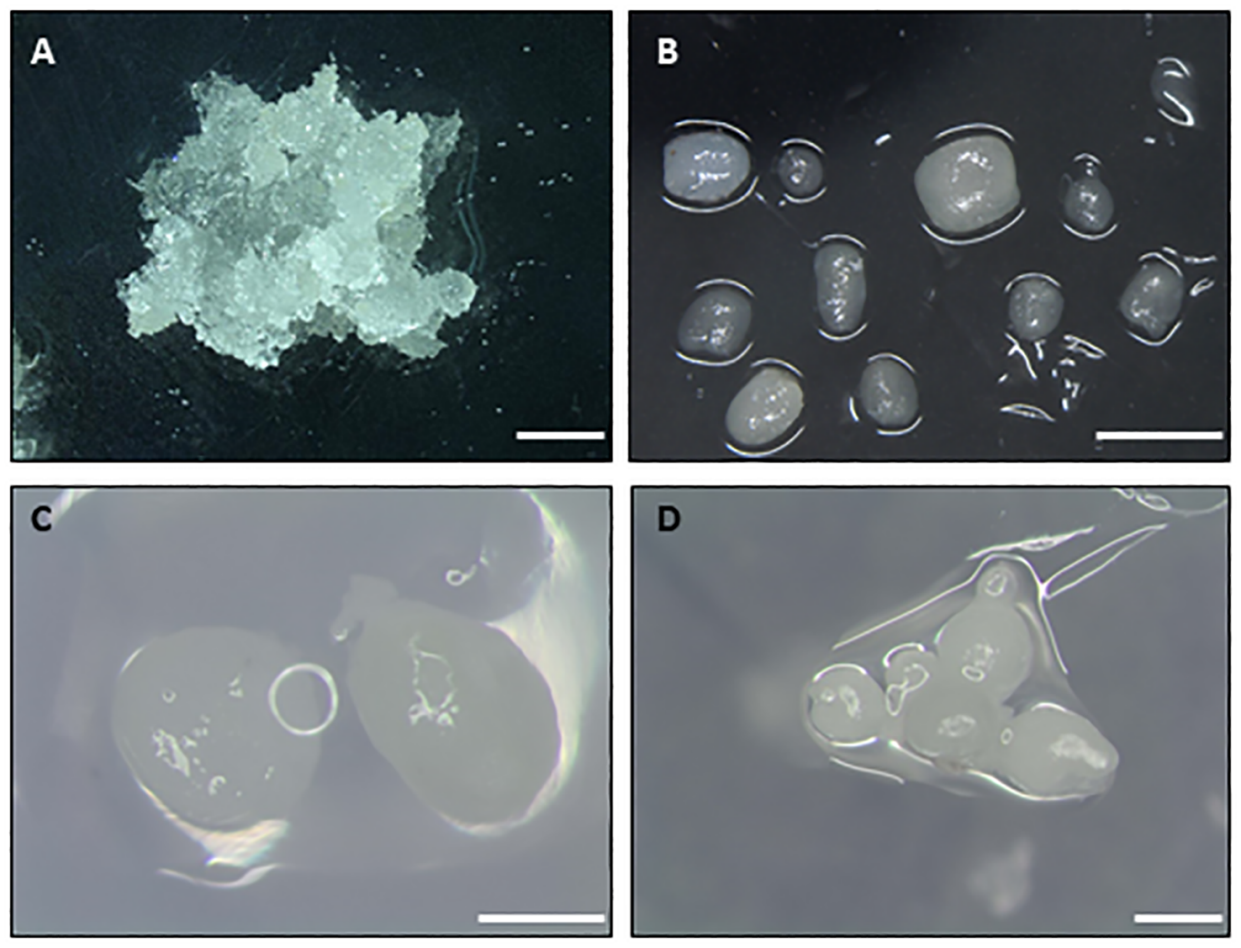
Types of initial explants used to cryopreserve embryogenic material of different woody species. **(A)** Embryogenic masses of maritime pine. **(B)** Nodular embryogenic structures of holm oak. **(C)** Globular embryos of European chestnut. **(D)** Groups of globular-torpedo stage embryos of cork oak. Bar 1 mm.

One important question to consider before the cryopreservation of embryogenic material is the selection of the initial explant used (**Figure 1**, first step). Indeed, the final response (i.e., success) to cryopreservation of embryogenic material varies widely in comparison with that of dormant buds, shoot tips and embryonic axes in which a shoot is obtained after LN storage. Different types of embryogenic explants are mentioned in the literature, including embryogenic callus, embryogenic masses, embryogenic tissues, embryogenic cell suspensions, proembryogenic masses (PEMs), nodular embryogenic structures, polyembryoids, and somatic embryos -usually as clumps or clusters (Corredoira et al., 2017a). The age, physiological state and the size must be considered. The use of young embryogenic cultures to isolate the initial explant is essential for successful recovery of the material after storage in LN. In addition, the selection of the developmental stage of somatic embryos used is another crucial aspect (Engelmann, 2011a), as undifferentiated stages or early embryo developmental stages (**Figure 2**) are the most appropriate explants (summarized as “key factors” in **Figure 1**).

## 3 Preconditioning treatments

Preconditioning refers to the manipulation of the embryogenic cell or tissue cultures before the cryoprotection treatment, which increases their survival and recovery after cooling in LN. The most common preconditioning treatments include cold hardening and treatment with osmotic agents or anti-stress compounds (**Figure 1**, step 2). Cold hardening involves exposing tissues to a low-temperature acclimatization regime prior to freezing (Reed, 1989; Benson, 2008b). For example, the cultures are maintained for a few days or weeks at lower temperatures than usual or under conditions of shorter daylight hours. However, it is a key factor that these hardening times are adjusted for each species or genotype (**Figure 1**). Cold hardening is widely used in the cryopreservation of shoot tips but is rarely used with embryogenic cultures.

Osmotic agents are used as a preconditioning treatment with the aim of reducing the explant’s water content before freezing (Hoekstra et al., 2001). In most published cryopreservation protocols, the embryogenic explants are cultured on media or solutions containing osmotically active agents such as sugars and sugar alcohols, usually at room temperature (Corredoira et al., 2017a). These compounds stimulate the accumulation of protective sugars, stabilizing membranes and proteins during the process of water loss (Dumet et al., 1993; Carpentier et al., 2007). With embryogenic cultures, preconditioning on sugar media is frequently employed in vitrification or encapsulation-dehydration procedures, and high concentrations of sucrose (ranged from 0.2 to 1M in fixed or increased in increments) are commonly added. Successful examples of this procedure are the cryopreservation of somatic embryos of cocoa (*Theobroma cacao* L.) (Fang et al., 2004), oil palm (*Elaeis guineensis* Jacq.) (Dumet et al., 1993), black alder (*Alnus glutinosa* (L.) Gaertn.) (San José et al., 2015) or holm oak (*Quercus ilex* L.) (Martínez et al., 2022). However, it is a key factor to adjust the concentration, time and conditions during the exposition to osmotic compounds for each species or genotype (**Figure 1**). A similar premise involves preculturing explants with antistress compounds such as abscisic acid (ABA) or amino acids as proline or glutamine. It is known that low concentrations of ABA induce tolerance to desiccation and cooling (Edesi et al., 2020). For instance, the preculture of embryogenic cultures of horse-chestnut (*Aesculus hippocastanum* L.) with 0.75 µM ABA and slow cooling had the best embryo recovery rates (Jekkel et al., 1998). In Norway spruce (*Picea abies*, (L.) H. Karst.) embryogenic tissue preconditioned with both sucrose and ABA was more effective than treatment with sucrose alone (Hazubska-Przybył et al., 2013).

## 4 Cryoprotection procedures

As indicated in the Introduction section, before embryogenic cells and tissues are exposed to LN, they must be protected from the formation of lethal intracellular ice. Diverse approaches for cryoprotection are deployed depending on the use of cryoprotective agents (**Figure 1**, step 3) and the cooling rate applied (**Figure 1**, step 4).

### 4.1 Classical (slow) cooling methods

The observation of a natural tolerance to freezing environments in overwinter organisms and the protective power of chemical substances like glycerol and dimethylsulphoxide (DMSO) in animal cells, led from the late 1950s to the early 1970s to the first plant cryopreservation developments in dormant and cold hardened buds of mulberry (*Morus* sp.), willows (*Salix* sp.) and poplars (*Populus* sp.) (Sakai, 1960), and in cell suspensions of flax (*Linum usitatissimum* L.) and carrot (*Daucus carota* L.) (Quatrano, 1968; Latta, 1971). These experiences constituted the basis of the so called classical (or slow) cooling methods (Panis, 2019). Essentially, the cryoprotective effects of the classical (slow) cooling methods are based on two principles: (1) the freezing of extracellular water leading to a non-lethal (and partial) freeze dehydration of intracellular contents, and (2) the subsequent vitrification (or formation of a glassy state) of the intracellular contents. To achieve these principles cells or tissues are firstly cooled down to temperatures between -30°C and -40°C in a slow manner and then cooled relatively fast (hundreds of °C/sec) to LN temperatures (generally -196°C). Although slow cooling rates of 1°C/min are commonly used (e.g., those provided by devices such as Mr. Frosty®, Nalgene), it is often a key factor to adjust the slow cooling rate for each species or genotype via programmable freezers (**Figure 1**, step 4). The use of chemical agents in some protocols are essential to further protect cells from osmotic damage and/or ice formation and growth that arise during the cooling steps of the classical (slow) cooling methods (e.g., Sakai, 1960; Panis, 2019). In this case, as with the vitrification solutions, temperature and time of exposition must be adjusted (**Figure 1**, step 3).

Nowadays classical (slow) cooling methods are implemented for the large-scale cryopreservation of diverse plant genetic resources. For example, woody horticultural temperate species such as *Malus* sp., *Pyrus* sp. or *Morus* sp. are routinely banked through the cryopreservation of their winter dormant buds (Fukui et al., 2011; Jenderek and Reed, 2017; Höfer and Flachowsky, 2023) and different successful reports suggest the possibility of its application to the dormant buds of many other clonal crops (Tanner et al., 2021). This technique has also great potential for the extensive cryopreservation of dormant buds of woody forest temperate species like birch (*Betula* sp.) or *Ulmus* sp. (Välimäki et al., 2021; Endoh et al., 2023) and it is potentially applicable to buds of some tropical crops when the technique and the cold hardiness of the plants are optimized (Atmakuri et al., 2009; Tanner et al., 2021). Classical (slow) cooling techniques are also broadly used for the long-term cryopreservation of embryogenic material of a wide range of unorganized plant tissues (Panis, 2019). Examples include embryogenic cell suspensions and/or embryogenic calli of banana (*Musa* sp.), *Abies* sp. and *Pinus* sp. (Panis et al., 1990; Salaj et al., 2007; Salaj et al., 2010). In these cell types, the methods employed typically require, as previously mentioned, the preculture of the cells in cryoprotective solutions that may include sugars such as sucrose or mannitol, amino acids such as proline, DMSO or/and glycerol.

### 4.2 Vitrification-based methods

The lack of success of the classical (slow) cooling methods in some non-hardy cultured plant cells, and the relatively technical complexity of the method (i.e., the use of programable freezers), led to Uragami et al. (1989) to adopt the first “vitrification-based” procedures in plant tissues. These procedures were initially developed for human cells and mouse embryos to avoid the formation of lethal or disruptive extracellular (and intracellular) ice and overcome the failure of the cryopreservation of these systems (Rall and Fahy, 1985; Takahashi et al., 1986). The cryoprotective effects of the procedure is based on the supercooling capacity of highly concentrated solutions of cryoprotective agents. They can remain liquid at very low temperatures (around -70°C) and become so viscous that finally solidify into a metastable glass (i.e., vitrify) without ice formation at a practical cooling rate (Rall and Fahy, 1985). Vitrification in these first reports was accomplished by the fast cooling of the cells and embryos after their exposure to a highly concentrated and low toxic vitrification solutions (VS) such as VS1, which contained 20.5% (w/v) DMSO, 15.5% (w/v) acetamide, 10% (w/v) propylene glycol and 6% (w/v) polyethylene glycol (Rall and Fahy, 1985). Uragami et al. (1989) performed their experiments in asparagus cultured cells and somatic embryos which were cryoprotected by the first Plant Vitrification Solution (PVS1), a mixture of 22% (w/v) glycerol, 15% (w/v) ethylene glycol, 15% (w/v) propylene glycol and 7% (w/v) DMSO in MS medium (Murashige and Skoog, 1962) containing 0.5 M sorbitol. This solution was later on modified and adapted to diverse cells and tissues (Zamecnik et al., 2021), and is nowadays massively used in the cryopreservation of plant germplasm, generally in the forms of PVS2 (typically, 30% (w/v) glycerol, 15% (w/v) ethylene glycol, and 15% (w/v) DMSO in MS medium containing 0.4 M sucrose) (Sakai et al., 1990) or PVS3 (typically, 50% (w/v) glycerol and 50% (w/v) (Nishizawa et al., 1993) sucrose in water or MS-based medium).

There are several plant vitrification-based procedures that differ in the exposition of cells and tissues (i.e., explants) to PVS and the cryogen (LN): vitrification, encapsulation-vitrification, droplet-vitrification and V-cryoplate (Sakai and Engelmann, 2007; Panis, 2019; **Figure 1**, step 3). In all four methods, the exposition of the explants to the PVS and LN is typically preceded by their preculture on sucrose-enriched medium and a subsequent treatment (‘loading’) with a solution (aka ‘loading solution’, LS) containing commonly 2 M glycerol + 0.4 M sucrose (Sakai and Engelmann, 2007; Panis, 2019). Moreover, when the explants are removed from LN, they are rewarmed quickly and subsequently exposed to a 1.2 M sucrose-enriched culture medium to favor the removal (‘unloading’) of the cryoprotective agents before they are moved to the appropriate *in vitro* culture conditions (Sakai and Engelmann, 2007). In the so called “vitrification” method, precultured explants are directly exposed to PVS for the appropriate period of time, which is species and explant dependent, followed by the immersion of a cryovial topped up with fresh PVS in which the cells or tissues as suspended in LN (Sakai et al., 1990). The exposition to PVS is a key factor which must be determined empirically (**Figure 1**, step 3). Similarly, in the “encapsulation-vitrification” method, the samples, that have been precultured, are exposed to LN within the PVS that is enclosed in a cryovial, with the difference that the cells and tissues are previously encapsulated in alginate beads (e.g., Pence et al., 2017). In the “droplet-vitrification” method, precultured explants are directly exposed to the PVS for the appropriate time, after which a small drop of the PVS containing the explant is placed on top of an aluminum foil strip that is rapidly plunged in LN (Pennycooke and Towill, 2000; Panis et al., 2011). This technique is widely used and has allowed a significant increase in the success of cryopreservation when compared to other plant cryopreservation methods (e.g., Panis et al., 2011; Vollmer et al., 2016; Pence et al., 2017). Some authors have even suggested that represents as “the first generic cryopreservation protocol for organized plant tissues” (Panis et al., 2011). Finally, in the V-cryoplate (V for vitrification), the plant tissue is encapsulated in small alginate droplets that are attached to a commercially available aluminum plate that fits into a 2-mL cryotube. Here, the explants are sequentially exposed to the LS and PVS, and then plunged in LN (Yamamoto et al., 2011). Cryo-storage and warming (thawing) are also performed with the V-cryoplates. This method can be considered as a hybrid method between droplet-vitrification and encapsulation-vitrification (Panis, 2019). It is being increasingly applied due to its high success rate and the easiness and speediness of specimens’ handling, as only the cryo-plates are manipulated.

### 4.3 Desiccation based methods

Physical (air) drying, to limits tolerated by the explants, is the easiest way to concentrate the intracellular aqueous solution and reduce the fraction of water susceptible of freezing, thus, to cryoprotect the explants. Optimal drying of recalcitrant seed tissues (embryonic axes), shoot tips or embryogenic material (calli, somatic embryos, etc) is generally achieved when moisture contents of the explants is lowered to around 20-30% (fresh weight basis) (Panis, 2019; Ballesteros and Pritchard, 2020). However, the final moisture content is often a key factor that needs to be adjusted depending on the species (**Figure 1**, step 3). Then, explants are relatively rapidly cooled to LN temperatures, reducing the chance of ice formation and favoring the vitrification of the cell contents (Walters and Pence, 2021).

Two methods are used that mostly differ in whether the explants are dried “naked” (desiccation or air drying) or encapsulated in alginate beads (encapsulation–dehydration). Furthermore, the encapsulation–dehydration procedures often include a step in which the beads are incubated in a high concentrated solution of sucrose (0.75-2 M) for 12 h to 7 days, depending on the species and explant, before the desiccation step (Panis, 2019). Recently, Niino et al. (2013) have also developed the D-cryoplate (D for desiccation) method. This procedure is similar to the V-cryoplate, in which the plant tissue is encapsulated by alginate on commercially aluminum plates. However, instead of using PVS as in the V-cryoplate, the cryoprotection is achieved in the D-cryoplate by the desiccation of the explants under a dry environment or a dry air.

Desiccation-based procedures involve either the exposure of the explants to a dry environment within a desiccator containing a desiccant (generally activated silica gel, providing relative humidity (RH) <15%) (e.g., Sherlock et al., 2005), or the exposure of the explants to a dry air using diverse flows (Ballesteros et al., 2021). In the later approach, the flows used are typically the sterile air of a laminar flow cabinet (ambient RH, likely 30-60%), the flow of dry nitrogen gas pumped through fish tank air diffusers at 6 L/min (RH < 6%; as per Walker and Ernst Jr., 1930), or the air flow generated by a computer fan enclosed in a jar containing activated silica gel (RH<10%) (Ballesteros et al., 2021). In all these approaches, it is a key factor to adjust the desiccation time to achieve the targeted optimal moisture content (**Figure 1**, step 3).

Desiccation (air drying) procedures are typically used in the development of cryopreservation protocols for embryonic axes of recalcitrant seeds (Ballesteros et al., 2021). On the other hand, encapsulation–dehydration procedures have limited large-scale implementation due to the sensitivity of diverse explants and species to high concentrations of sucrose and the large desiccation needed (Panis, 2019). Nonetheless, encapsulation–dehydration procedures are highly successful in the cryopreservation of fern gametophytes (Ballesteros and Pence, 2018). The D-cryoplate method is not extensively used but has been optimized for the routine cryopreservation of diverse woody species and tissues, including polyembryonic masses of date palm (*Phoenix dactylifera* L.) (Salma et al., 2014).

## 5 Rewarming

Rewarming (aka “thawing”) of the cryopreserved samples is also a critical step in all cryopreservation procedures (**Figure 1**, step 5), regardless of the type of explant (Bettoni et al., 2021). During rewarming, generally at temperatures >-130°C, the cryopreserved samples devitrify (i.e., the glassy state is lost) and ice crystals can be formed *de novo* or grow from small ice nucleus formed upon cooling, resulting in the formation of lethal ice crystals (Benson, 2008a). To prevent this ice recrystallization, the material must be rapidly rewarmed. However, a key factor of this step is to find the optimal rewarming temperature and time (**Figure 1**, step 5). For example, the temperature of the warming environment ranges between 25°C (room temperature) and 42°C, depending on the protocol. Also varies the way explants are exposed to the warming environment. For example, in some protocols, the whole vials containing the explants are plunged directly into a warm water bath (37-42 °C) for 2-4 min. Alternatively, the vials with the explants (e.g., alginate beads containing encapsulated tissues) are warmed at room temperature. In other protocols, alginate beads, but also aluminum foils or plates from the droplet or cryoplates methods, are taken out from the cryovial and directly immersed in warm (25-42 °C) liquid medium placed into a Petri dish. Finally, after rewarming, is also frequent in vitrification and encapsulation-vitrification methods to immerse the embryogenic material in an ‘unloading’ solution with 0.3–1.2 M sucrose for a short period (Corredoira et al., 2004; San José et al., 2015; O’Brien et al., 2016a)

## 6 Assessment of the efficacy of cryopreservation

**Figure 3:**
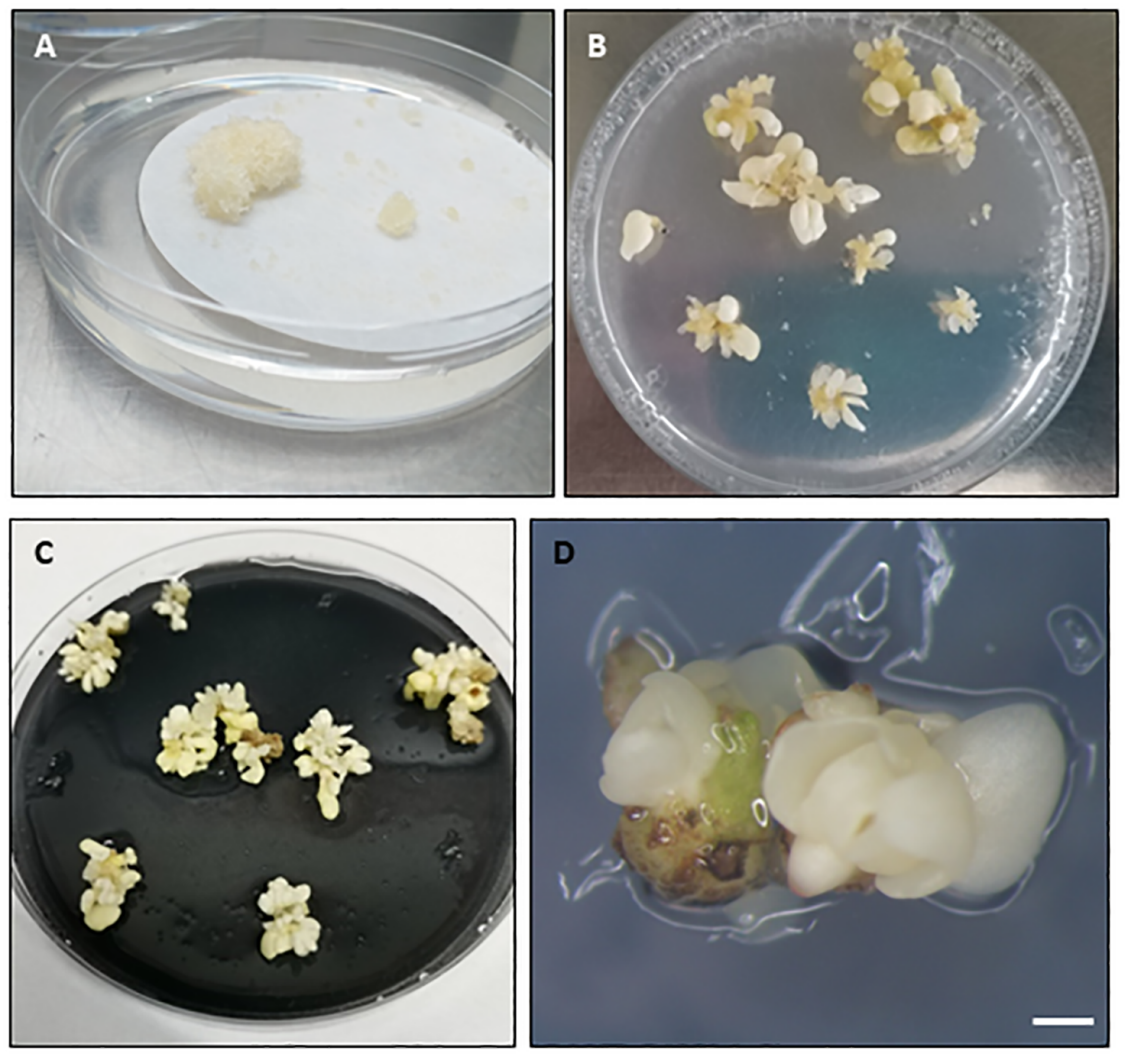
Embryogenic response in different woody species observed after liquid nitrogen storage and culture on recovery medium. **(A)** Embryogenic masses recovery in maritime pine. **(B–D)** Somatic embryo recovery in holm oak **(B)**, white oak **(C)** and black alder **(D)**. Embryogenic masses in **(A)** were cryopreserved following a slow cooling method, while the embryogenic tissues in **(B–D)** were cryopreserved following vitrification after cryoprotection with PVS2. In **(B)** the cryopreserved explants were nodular embryogenic structures while in **(C, D)** groups of somatic embryos. **(A–C)** Diameter Petri dish, 90 mm. **(D)**, Bar=1mm.

Recovery of viable somatic embryos is the final goal of cryopreservation procedures with embryogenic tissues (**Figure 1**, step 6). First, thawed explants are usually cultured to Petri dishes containing filter paper placed over embryo recovery/proliferation medium, for 24-48 h, during which the explants release toxic compounds generated during the stress caused by the cryopreservation and thawing process. The explants are then cultured *in vitro* to assess the efficacy of the cryopreservation procedure. Many different terms or parameters are used to define the response of the explant to LN storage, including viability, survival, growth, regrowth, embryo recovery and callus formation. However, for a protocol to be considered effective, viable somatic embryos or embryogenic callus capable of generating new somatic embryos must be obtained after recovery from LN (**Figure 3**). Moreover, for clonal explants, it is recommended that a recovery rate greater than 20-30% (Vollmer et al., 2016) or 40% (Reed, 2001) should be obtained for a particular embryogenic line (i.e accession) to be considered safely cryopreserved.

## 7 Cryopreservation of embryogenic material in woody species

### 7.1 Cryopreservation in conifer species

In conifers, somatic embryos are obtained through indirect SE, or in other words, with an intermediate multiplication state of proembryogenic masses (Egertsdotter, 2019). Although there are some reports on cryopreservation of somatic embryos, is at the abovementioned callus state when embryogenic tissues are cryopreserved in order to retain their capacity to produce somatic embryos in the future (Lelu-Walter et al., 2013). Since the first reports on SE (Chalupa, 1985; Hakman et al., 1985), this propagation method has been mainly developed in species with economical relevance. However, in the last years it has also been developed for endangered species (Ahn and Choi, 2017; Ahn et al., 2019; Maruyama and Hosoi, 2019; Jouini et al., 2023).

As mentioned in the Introduction, in conifers the refinement of SE technique has contributed to the development of the forestry industry through multivarietal forestry (Park et al., 2016). Reports on SE from adult conifers are scarce (Trontin et al., 2016), and SE is usually initiated from young tissues where the characteristics of the future tree are not shown yet, so a previous testing of somatic plantlets is required to determine which clones are of interest. This is why well refined cryopreservation protocols are pivotal for the implementation of multivarietal forestry.

In the case of threatened species, the added value of having efficient cryopreservation methods to preserve biodiversity is evident. Developing SE and cryopreservation protocols leaves time for research to find solutions to the problems affecting these species and somatic seedlings can be used for reforestation or to establish new populations (Ma et al., 2012). These technologies offer an advantage over other tissue culture methods such as organogenesis for several reasons, the initial explant for SE is an immature zygotic embryo which contributes to genetic diversity much more than cloning the donor tree genotype and once this propagation technique and cryopreservation protocols are refined, the number of plantlets obtained is generally higher.

Among the different plant cryopreservation methods available, the slow cooling method (see section 4.1) is used for the vast majority of conifer PEMs since the first reports back in the eighties of the last century (Gupta et al., 1987; Kartha et al., 1988). Summarizing, the steps to carry out this procedure are: preconditioning of tissues, cryoprotection, slow cooling to a temperature close to -40°C, and rapid immersion in LN (Engelmann, 2011b).

In the preconditioning treatment, PEMs are usually pregrown in semisolid (*Pinus sylvestris* L., Latutrie and Aronen, 2013) or liquid media (*Picea pungens* Engelm., Cao et al., 2022) containing increasing concentrations of cryo-protective substances as sugars (sucrose (*Pinus elliottii* x *P. caribaea* Nunes et al., 2017) or maltose (*Pinus pinaster* Aiton, Cano et al., 2018), or sugar alcohols [sorbitol (*Abies alba* Mill., Salaj et al., 2022)] or mannitol (*Araucaria angustifolia* (Bertol.) Kuntze, Fraga et al., 2016); the duration of this preculture varies from hours (*Pinus nigra* J.F. Arnold, Salaj et al., 2012) to days (*P. pinaster*, Marum et al., 2004) depending on the protocol (**Supplementary Material 1**).

Following the preconditioning treatment, the tissue is exposed to a cryoprotective solution usually composed by cryoprotective penetrating substances as DMSO (added to a final concentration of 5 to 15%, Panis and Lambardi, 2006), or a mixture of various penetrating [e.g., proline and glycerol, *Cryptomeria japonica* D. Don (Taniguchi et al., 2020)] and non-penetrating [e.g., high molecular mass PEG, *Picea abies* (Varis et al., 2022)] substances. This step lasts from minutes (*Picea sitchensis* (Bong.) Carr., Gale et al., 2007) to hours (*Abies alba*, Krajňáková et al., 2013) and is performed in an ice bath (at 4°C) to avoid toxic effects of DMSO (**Supplementary Material 1**). The toxicity of DMSO has been reported in some studies indicating an increase in benzene metabolism and other aromatic hydrocarbons toxicity (Kaya and Souza, 2016 and references therein).

After tissue conditioning with cryoprotectant, the slow cooling is made by means of cooling boxes (such as “Mr. Frosty®”), or programable freezers. Again, the duration of this step usually varies from minutes (Álvarez et al., 2012) to 24 hours (Marum et al., 2004) depending not only on the species but on the protocol. Then, the cryovials are plunged into LN. This methodology has been also used to store embryonal masses of *P. radiata* and *P. attenuata* x *P. radiata* at -80°C (Montalbán and Moncaleán, 2017; Montalbán et al., 2021).

Thawing procedures usually require a rapid immersion of cryovials into a warm water bath (around 40 °C) for a few minutes, until the solid ice core has melted (Ford et al., 2000); if the cryoprotective solution contains DMSO, the cryovials are sometimes kept in ice after thawing (Mathur et al., 2003) to avoid its toxic effect, as mentioned above. After that, the cryoprotective solution is drained and the tissue is cultured directly in liquid or semisolid medium (Ahn et al., 2018) or pre-rinsed with liquid medium (Maruyama et al., 2000).

The culture media employed for tissue recovery can be those used for initiation or proliferation at SE initial stages (Castander-Olarieta et al., 2022) or media with the same content of sugar or sugar alcohol as in the pre-treatment of tissues but in reverse order (Varis et al., 2017). Some authors have found a nurse culture helpful to regenerate the thawed tissues (Hargreaves et al., 2002).

The success of a protocol can be evaluated in terms of tissue regrowth and maturation ability of the regenerated embryogenic cell lines. This last parameter is important because although the biological processes are suspended when tissues are cryopreserved, they can be damaged during conditioning, freezing or thawing, resulting in anembryogenicity loss (Lambardi et al., 2018).

A few reports on embryogenic tissue cryopreservation through vitrification are also available mainly for *Picea* embryogenic (*Picea mariana* (Mill.) Britton, Sterns & Poggenburg, Touchell et al., 2002) and non embryogenic tissues (*Picea abies*, Viljamaa et al., 2018). In these species, PVS2 solution (Sakai et al., 1990) or its modifications were used as cryoprotectant. This solution has also been tested in *Araucaria angustifolia* (Demarchi et al., 2014) and *Larix kaempefri* × *L. gmelinii* embryo-genic tissues (Ma et al., 2023); however, in these cases, the samples were cooled slowly before LN. In other species, vitrification *versus* slow cooling methods have been compared for cryopreservation of embryogenic tissues (Lambardi et al., 2018) and, as a general trend, the slow-cooling method has given the best results.

Another cryopreservation method, by means of desiccation over silica gel (see section 4.3) of the PEMs has proven to be successful in *Picea* (Hazubska-Przybył et al., 2010; Hazubska-Przybył et al., 2013). In this sense, desiccation (Percy et al., 2001), together with vitrification or encapsulation (Gale et al., 2008) has been tested for cryopreservation of somatic embryos at different developmental stages. After desiccation and LN storage, somatic embryos from different species were able to germinate, e.g., *Chamaecyparis pisifera* (Siebold & Zucc.) Endl. (Maruyama et al., 2003), *Picea glauca* (Moench) Voss (Percy et al., 2001), *Picea mariana* (Bomal and Tremblay, 2000), *Pinus monticola* Douglas ex D. Don (Percy et al., 2000); or to give rise to PEMs (Gale et al., 2008).

### 7.2 Cryopreservation in fruit species

To date, cryopreservation in fruit trees has been reported for embryogenic material of *Olea europea* L., *Persea americana* Mill., *Theobroma cacao* Mill., *Vitis vinifera* L. and species of the genera *Citrus* and *Coffea* using different tissues as starting explants and cryopreservation techniques (**Supplementary Material 2**). In general, the results obtained by the slow cooling methods developed for olive, avocado and species of the genera *Citrus* and *Coffea* have been improved by the application of vitrification-based techniques, mainly vitrification and droplet-vitrification.

For example, in *Citrus*, slow cooling has been applied to embryogenic calli and cell suspensions of a variety of species, including *C. sinensis* and *C. deliciosa* (Kobayashi et al., 1990; Aguilar et al., 1993; Marín et al., 1993; Engelmann et al., 1994; Pérez et al., 1997; Olivares-Fuster et al., 2000; Singh et al., 2007). No preconditioning treatments were carried out before cryoprotection. Cryoprotectant solutions normally contained DMSO and sucrose at variant concentrations, although the sugar complement not always was present. According to Engelmann et al. (1994), increasing the DMSO concentration to 10-15% in the cryoprotective medium generally improved recovery after freezing. Slow cooling was carried out by using programmable freezers, “Mr. Frosty®”, or methanol cooling baths. Both the protocol executed and the genotype greatly affected viability after freezing. Cryopreservation of *Citrus* embryogenic cultures have also been successfully carried out by using the vitrification procedure (Sakai et al., 1990; Sakai et al., 1991; Hao et al., 2002). In all cases, cells were exposed to PVS2 for short times, mainly at 25°C or room temperature. As in the slow cooling, no preconditioning and/or loading treatments were applied before incubation in PVS2. Explants were directly immersed in LN. All genotypes survived cryopreservation with survival rates higher than 80%.

Somatic embryos of coffee species, *Coffea arabica* L. and *Coffea canephora* Pierre ex A. Froehner, were cryopreserved by using slow freezing (Dereuddre et al., 1994). Previous to DMSO treatment, somatic embryos were incubated in culture media with 0.3-0.5 M sucrose. Survival rates obtained after rewarming (57% and 28% in *C. arabica* and *C. canephora*, respectively) significantly improved when embryos were kept in darkness for 3 weeks during recovery. In parallel, Tessereau et al. (1994) cryopreserved *Coffea canephora* somatic embryos by desiccation. As coffee somatic embryos did not survive below 92% relative humidity, various hardening treatments were tried to improve desiccation tolerance. The best results after freezing (80-90% viability) were achieved following a two-step hardening treatment consisting of incubation in 0.15 M sucrose and 1 µM ABA for 4 weeks, then in 0.44 M sucrose and 1 µM ABA for 2 weeks. Later, Mycock et al. (1995) compared a cryopreservation protocol using cryoprotectants and partial drying before freezing with other one consisting of partial dehydration and rapid freezing. Similar survival percentages (66-72%) were achieved irrespective of the procedure. Both, cooling rate and drying were important factors for successful cryopreservation. In fact, dehydration only was an effective pretreatment for cryopreservation when it was followed by rapid cooling.

Slow cooling and vitrification methods were used by Benelli et al. (2001) for cryopreservation of olive somatic embryos at different developmental stages. Higher post-thaw regrowth (38%) was achieved by using the vitrification procedure after dehydration in ice-cold PVS2 for 90 min. Sánchez-Romero et al. (2009) optimized cryopreservation of olive embryogenic cultures by comparing three methods: slow cooling, classical vitrification and droplet-vitrification on aluminium foil strips. Better results were obtained by using the vitrification-based techniques after dehydration in PVS2 at 0 °C for 60 min, with 100% recovery reached in both cases. Nevertheless, significantly higher regrowth rates were found in samples following the droplet-vitrification method. Long-term preculture in basal medium with 0.4 M sucrose significantly affected the initial explants response after cryopreservation. Lynch et al. (2011) applied a slow cooling procedure to cryopreserve clumps of somatic embryos. After 3-day preconditioning treatment in solid medium with 0.75 M sucrose, somatic embryos were cryoprotected for 1 h on ice with a solution containing 1 M sucrose, 0.5 M DMSO, 0.5 M glycerol and 0.009 M proline. Subsequently, they were cooled in a programmable freezer with an equilibration hold at 0°C for 10 min, a cooling rate of 0.5 °C min-1 to −35 °C and a hold at −35 °C for 35 min, before plunging into LN. Osmotic preconditioning treatment significantly affected regrowth of cryopreserved embryos (assessed as fresh weight gain), with the best results (34.6%) found after a 0.75 M sucrose pretreatment. Droplet-vitrification was also utilized to cryopreserve olive somatic embryos, structurally very different to embryogenic calli (Bradaï et al., 2017). After 30 min dehydration in PVS2, 60% of samples resumed embryogenesis. Culture conditions of somatic embryos had a determinant influence on their subsequent response to cryopreservation. Preculture in liquid medium supplemented with 0.2 M sucrose for 28 days significantly improved cryopreservation, with 90% of explants resuming embryogenesis after rewarming (Bradaï et al., 2023).

Avocado embryogenic cultures were firstly cryopreserved by Efendi (2003). After freezing, very variable recovery values (from 0 to 80%) were obtained depending on the cultivar. The process was subsequently optimized by Guzmán-García et al. (2013). For this purpose, three cryopreservation protocols, a slow cooling method, the vitrification protocol, and droplet-vitrification on aluminium foil strips (see sections 4.1 and 4.2), were applied to two embryogenic lines, representative of the SE- and PEM-type cultures obtained in this species (Márquez-Martín et al., 2012). The results obtained with the slow cooling procedure were greatly influenced by the embryogenic line, whereas the vitrification-based procedures provided high recovery percentages (77.8-100%), regardless of the genotype. Although similar results in terms of survival and recovery were obtained with the vitrification techniques tested, slightly higher regrowth rates were recorded in samples cryopreserved by using droplet-vitrification. Vitrification and droplet-vitrification procedures optimized by Guzmán-García et al. (2013) were afterwards applied to other avocado cultivars, also reporting better results with the droplet-vitrification method (O’Brien et al., 2016b; O’Brien et al., 2016a). Modifications in the LS treatment regarding sucrose concentration and exposure time contributed to improve recovery in cultivars exhibiting low survival percentages when directly applying the protocol of Guzmán-García et al. (2013) (O’Brien et al., 2016a). Slightly lower viability rates were obtained after 12-month LN exposure (O’Brien et al., 2016a; O’Brien et al., 2016b).

Encapsulation methods have been used to cryopreserve embryogenic cultures of olive (Shibli and Al-Juboory, 2000), cocoa (Fang et al., 2004), *Citrus* spp. (González-Arnao et al., 2003), and gravepine (*Vitis* sp.) (González-Benito et al. (2009). In gravepine, González-Benito et al. (2009) encapsulated embryogenic cells in alginate beads. After preconditioning in liquid medium supplemented with 1 M sucrose for 4 days and desiccation for 2-4 hours in a laminar flow bench, explants were plunged in LN. All cryopreserved samples exhibited vigorous growth (100% regrowth) eight weeks after thawing. In olive, cotyledonary somatic embryos (1-2 mm in size) were cryopreserved by both encapsulation-dehydration and encapsulation-vitrification. Higher regrowth rate (54%) was achieved after dehydration in PVS2 at 0 °C for 3 h. Pretreatment of the embryogenic calli at 30 °C for one day before encapsulation slightly increased regrowth of cryopreserved embryos up to 58% (Shibli and Al-Juboory, 2000). In cocoa, Fang et al. (2004) developed an encapsulation-dehydration procedure for somatic embryos, optimizing embryo development stage, cryoprotectants (ABA and sugar), and duration of osmotic and evaporative dehydration. A post-cryo survival ranging from 25-72% was achieved after preculture with 0.75-1 M sucrose for 7 days and silica exposure for 4 h (16% moisture content in bead). However, slightly better results (42.9-74.5% post-cryostorage survival) were obtained on different cocoa genotypes by Adu-Gyamfi and Wetten (2020; Adu-Gyamfi and Wetten, 2012) by vitrification. This method required a 5-day preculture of the somatic embryos on culture medium with 0.5 M sucrose, and subsequent exposure to cold PVS2 during 60 min. González-Arnao et al. (2003) cryopreserved *Citrus* embryogenic tissues by encapsulation-dehydration, following the basic protocol of Fabre and Dereuddre (1990). In this procedure, alginate-coated somatic embryos were pregrown in liquid medium with 0.75 M sucrose for 1 day and dehydrated in a laminar flow cabinet down (20-25% of moisture content in the beads) prior to immersion in LN. 76-100% survival was achieved after thawing at room temperature. More recently, Souza et al. (2017) cryopreserved embryogenic calli of *C. sinensis* using a modified encapsulation technique on an aluminium cryoplate. In this method, callus cells encapsulated in alginate beads were placed on the bottom of the open aluminium foil through and covered with 80 μL of PVS2. After incubation on ice for 30 min, beads in PVS2 were well packed in the double aluminium foil troughs and rapidly plunged in LN. A survival rate of 88.7% was achieved after rewarming by immersing the frozen aluminium foil troughs in unloading solution at room temperature for 20 min.

### 7.3 Cryopreservation in deciduous forest species

Deciduous forests include important tree species such as alder (*Alnus* sp.), ash (*Fraxinus* sp.), aspen (*Populus* sp.), beech (*Fagus* sp.), birch (*Betula* sp.), chestnut (*Castanea* sp.), elm (*Acer* sp.), maple (*Acer* sp.), oak (*Quercus* sp.) or walnut (*Juglans* sp.). In deciduous species, clumps or groups of somatic embryos are the most frequently used explant (**Supplementary Material 3**). The physiological/developmental stage of somatic embryos is considered a crucial aspect and somatic embryos at early developmental stages (i.e., PEMs, globular, heart or torpedo stages) yield the highest recovery rates after storage in LN (Corredoira et al., 2014). For example, de Boucaud and Brison (1995) established that in walnut somatic embryos at the early globular stage were more tolerant to freezing than other developmental stages. Likewise, small groups of globular-or heart-stage somatic embryos of cork oak (*Quercus suber* L.) yielded higher embryo recovery rates than cotyledonary-stage embryos (Valladares et al., 2004). In holm oak (*Q. ilex* L.) long-term cryostorage was only possible when nodular embryogenic structures (aka PEMs) were used instead of globular embryos (Martínez et al., 2022 *versus* Barra-Jiménez et al., 2015). More often somatic embryos are preconditioned by the culture on media supplemented with high concentrations of sucrose or sorbitol for 1 to 7 days (**Supplementary Material 3**). Exceptionally ABA preculture (Jekkel et al., 1998) and cold hardening have also been applied as preconditioning treatments (de Boucaud and Brison, 1995; Lambardi et al., 2005)

Cryoprotection of embryogenic explants is mainly restricted to treatment by the vitrification technique or by desiccation in a laminar airflow cabinet (**Supplementary Material 3**). In our knowledge, only two examples of applications of the encapsulation–dehydration procedure (Scocchi et al., 2007; Fernandes et al., 2008) and one example of droplet-vitrification (Shin et al., 2012) have been published to date. In almost all studies comparing vitrification and desiccation procedures, vitrification produced the best embryo recovery frequencies. For instance, in *Quercus robur* L. (pedunculate oak), Martínez et al. (2003) reported embryo resumption rates of about 70% using vitrification procedure, whereas the recovery rates were 56% when clumps of somatic embryos were preconditioned on sucrose-supplemented media before desiccation in a laminar airflow cabinet (to 24–34% water content). Likewise, after applying a similar desiccation procedure to that used in pedunculate oak, only 33% of European chestnut (*Castanea sativa* Mill.) embryos recovered their embryogenic capacity, relative to the recovery rate of 68% obtained with vitrification treatment (Corredoira et al., 2004). In vitrification procedures, the cryoprotective solutions are PVS2 or DMSO (at different concentrations) in combination with sucrose or glycerol or alone (**Supplementary Material 3**). The exposure time to the vitrification solution and its temperature are the most evaluated factors (Martínez et al., 2003; Valladares et al., 2004; Corredoira et al., 2014; San José et al., 2015). Generally, the best exposure time to PVS2 was found to be 60–90 min at room temperature or 0 °C (**Supplementary Material 3**).

Direct immersion in LN of embryogenic explants is frequently used, but positive results have also been reported after slow cooling (Jekkel et al., 1998; Vendrame et al., 2001; Scocchi et al., 2007; Ozudogru et al., 2010; Yu et al., 2022). Rapid thawing approach is employed in most protocols, whereby the cryopreserved tissues are plunged into water at 40-42°C for 2-3 min (**Supplementary Material 3**). In the majority of species, the success of the cryopreservation procedure applied has been determined as embryo recovery, that is, as new embryo formation (**Supplementary Material 3**).

Among deciduous forest species important advances in the cryopreservation of somatic embryos were made in recent years in *Fagaceae* species (Ballesteros and Pritchard, 2020). Furthermore, it is important to highlight that in the case of many oak species, the embryogenic lines employed in cryopreservation experiments were induced from explants obtained from adult genotypes (Hernández et al., 2003; Toribio et al., 2004; Corredoira et al., 2012a; Martínez et al., 2017; Martínez et al., 2021). Embryogenic cultures of *C. sativa*, *Q. robur*, *Q. suber*, *Q. alba* L. (white oak) and *Q. ilex* have been successfully cryopreserved after undergoing vitrification-based procedures (**Supplementary Material 3**). The basic protocol consists of preconditioning somatic embryo clumps (isolated at early developmental stage) on multiplication medium with 0.3 M sucrose. After the preculture period, groups of embryos were incubated in cryovials with PVS2 solution during 1 hour (pedunculate oak and white oak) at room temperature or at 0°C (European chestnut and cork oak). Exceptionally, in holm oak nodular embryogenic structures were cryoprotected by applying a significantly shorter incubation period (15 min at room temperature) in PVS2 (Martínez et al., 2022). After this time, somatic embryo clumps were rapidly immersed in liquid nitrogen. Acceptable embryo recovery frequencies were reported, with percentages ranging from 57–92% in pedunculate oak (Martínez et al., 2003; Sánchez et al., 2008), 68% in European chestnut (Corredoira et al., 2004), 88–93% in cork oak (Valladares et al., 2004), about 54% for white oak (Corredoira et al., 2014), and 63% in holm oak (Martínez et al., 2022). In European chestnut, cork oak and holm oak, these procedures have also been successful applied to cryopreserve transgenic embryogenic lines obtained after coculture with *Agrobacterium tumefaciens* (Corredoira et al., 2007; Corredoira et al., 2012b; Cano et al., 2020; Cano et al., 2021). For example, in holm oak resumption rates of somatic embryos transformed with the thaumatin-like protein gene ranged from 43–77% (Cano et al., 2020). Similarly, the procedure established for European chestnut somatic embryos was also applied in the long-term conservation of transgenic lines that overexpress a thaumatin-like protein gene (Corredoira et al., 2012b) or a chitinase gene (Corredoira et al., 2016). The cryopreservation of these transformed embryogenic lines is thus of outmost value, as it allows their safe storage at reduced cost, while their tolerance to different pathogens is evaluated.

### 7.4 Cryopreservation in palm tree species

To date, the cryopreservation of embryogenic cultures of members of the palm family (*Arecaceae*) has been reported for *Bactris gasipaes* Kunth (peach palm), *Cocos nucifera* L. (coconut), *Elaeis guineensis* Jacq. (oil palm) and *Phoenix dactylifera* L. (date palm) and (**Supplementary Material 4**). In palm trees, the types of explants stored in LN are mainly PEMs, embryogenic callus, clusters/clumps of somatic embryos and polyembryoids (a specific embryogenic development stage in palms). Preconditioning treatment consists of cultivating the embryogenic tissues in the presence of sucrose at high concentration (from 0.3 to 1 M for 60 min to 7 days). The different cryoprotection methods used include vitrification, encapsulation-dehydration, desiccation, droplet-vitrification and cryoplate (V or D), although encapsulation-dehydration and droplet-vitrification are the most commonly used techniques (**Supplementary Material 4**). Explants are usually frozen by ultrarapid freezing by direct immersion in LN.

Among the aforementioned species, cryopreservation has mainly been investigated in oil palm and date palm (**Supplementary Material 4**). In oil palm, the low recovery rates (8.3-33%) initially obtained after preculture of explants on 0.75 M sucrose followed slow cooling or direct immersion in LN (Engelmann and Dereuddre, 1988; Engelmann, 1990), have significantly improved on in the last decade. Thus, Gantait et al. (2015) obtained a recovery rate of 68% after culture of isolated polyembryoids on preculture medium containing 0.5 M sucrose and cryoprotection of the material by droplet-vitrification before rapid immersion in LN. More recently, Palanyandy et al. (2020) used encapsulation-dehydration with clumps of somatic embryos, achieving 73% embryo recovery. In date palm, excellent results, in some cases with recovery rates higher than 90%, have been obtained with proembryogenic masses, which are usually preconditioned with 0.5 M sucrose and cryoprotected with droplet-vitrification (Fki et al., 2011; Salma et al., 2014), V cryoplate (Salma et al., 2014) or D cryoplate (Salma and Engelmann, 2017). Information about cryopreservation of coconut and peach palm is scarce. Welewanni et al. (2020) successfully cryopreserved coconut embryogenic callus for the first time by encapsulation-dehydration in silica gel. In peach palm, the highest recovery rates (52%) have been achieved by PVS3 and droplet-vitrification (Heringer et al., 2013).

## 8 Genetic stability analysis in cryopreserved embryogenic material

Somatic embryogenesis is the preferred protocol for forest tree propagation; hence, the assessment of the genetic stability of the regenerants is necessary. The main advantage of the SE over other vegetative propagation techniques relays on their capability of undergoing recurrent or cleavage embryogenesis, that allows establishing lines that can be maintained by periodical subcultures. This procedure, however, is labor-intensive and onerous, and in some conifer species affects further somatic embryo germination (Klimaszewska et al., 2016). Long-term subcultures can also be a source of genetic instability and somaclonal variation (Rodríguez López et al., 2010). For example, several reports have described somaclonal variation solely as a result of the induction, manipulation and regeneration of plants during the SE process (Isabel et al., 1996; Fourré et al., 1997; Burg et al., 2007; Marum et al., 2009; Henao-Ramírez et al., 2021). In addition, recent studies in Norway spruce demonstrated that shortened telomeres can result from prolonged *in vitro* culture, although embryo production was not found to be related with telomere length (Aronen et al., 2021). The cost saving associated with reducing subcultures, and the need of maintaining maturation competence, make cryopreservation a critical stage of the SE process to preserve the juvenility of lines while corresponding clones are tested (Klimaszewska et al., 2016). When tree performance has been evaluated, true-to-type selected varieties can be latter recovered and propagated from the cryostock.

### 8.1 Somaclonal variation in cryopreserved embryogenic material

Survival and regeneration of plantlets from cryopreserved explants is the main parameter determining the success of a cryopreservation protocol. However, somaclonal variation exhibited by these plants may be a serious constraint for the applicability of this technique for long-term conservation of genetic resources. For example, the use of shoot tips for cryopreservation is quite extended because they are more prone to maintain genetic stability. On the other hand, embryogenic tissue needs to be carefully manipulated to maintain stability (Li et al., 2018).

Somaclonal variation has been observed as a result of the whole culture-cryoprotection-regeneration process. Because metabolic processes are suspended until tissue retrieval from LN storage, minimal genetic instability is expected during cryogenic storage at –196°C (Kartha, 1985), despite the remote possibility of free radical formation or molecular damage due to ionizing radiation over long-term storage (Harding, 2004). However, during the cryoprotection-regeneration process, cells are forced to cope with physical, chemical and physiological stressful conditions, that could cause cryoinjury and genetic variations. For example, the stressful conditions imposed to plant cells during the cryopreservation protocol result in reactive oxygen species (ROS) production. Changes in ROS content and in ROS-scavenging related enzyme activity could be related to changes in gene expression, and this possibility has been studied during the cryopreservation of zygotic embryos and embryogenic calli of *Elaeis guineensis* (Wei et al., 2023). In addition, the common use DMSO as cryoprotectant at concentrations up to 10% is related to diverse damaging reactions (Harding, 2004). Cryoinjured cells will divide during regeneration and will differentiate new tissues and organs, which will transfer any genetic change to the new progeny. However, not all the stressful conditions imposed to plant cells during the cryopreservation protocol will result in a negative impact. For example, Wei et al. (2023) found that during a cryopreservation protocol, embryogenic tissue accumulated ROS scavenging enzymes that may increase cryotolerance to obtain a high survival rate. Thereby, a correct evaluation of the genetic integrity (true-to-type checking) of cryopreserved embryogenic cultures is crucial for conservation purposes.

Somaclonal variation may be manifested at the morphological, cytological, biochemical and molecular (nuclear and organellar genomes) level (Cyr and Klimaszewska, 2002). Then, the genetic stability of embryogenic cells during the cryopreservation process can be assessed by several methods, including flow cytometry, chromosome determination, molecular markers, and biochemical and phenotypic analyses (Sadat-Hosseini et al., 2019). From a molecular point of view, somaclonal variation has been described as an epigenetic alteration that can persist for many generations, therefore threatening the commercial viability of SE technology (Butiuc-Keul et al., 2016). This alteration comprises both genomic (large-scale deletions, variations in chromosome structure/number, and point mutations) and epigenetic changes (Wang et al., 2014).

The study of cryoinjury, genetic stability, dynamic and behavior of cryopreserved cells tissues or organisms have been referred to as Cryobiomics (Kaviani and Kulus, 2022). Those studies connect causal factors related to cryoinjury and loss of explant viability with the risk of genetic stability (Martinez-Montero and Harding, 2015). Recently, Popova et al. (2023) reviewed the physical and chemical factors affecting regrowth after cryopreservation and proposed factor combinations that may account for the recovery of cryopreservation-sensitive species. Genetic stability after and during cryopreservation of most important woody tree species is depicted in **Supplementary Material 5**.

### 8.2 Morphological (phenotypical) variation in cryopreserved embryogenic material

In general, regenerated plants display no morphological variation after cryopreservation (Harding, 2004). For example, cryopreservation did not modify the regeneration potential or the quality of the regenerated plants in olive (Bradaï and Sánchez-Romero, 2021), in grapevine (Wang et al., 2002; Vasanth and Vivier, 2011), in paradise tree (*Melia azedarach* L.) (Scocchi et al., 2007), and in *Citrus* sp. (Pérez et al., 1998). In contrast, phenotypic variability has been reported for cryopreserved somatic embryos-derived cocoa plantlets (Wetten et al., 2009). Evidences suggest that the nature of this variation is epigenetic (Adu-Gyamfi et al., 2016); that is, it comes from heritable changes in gene expression not associated with changes in the underlying DNA sequence. However, other authors reported no variability for some cultivars of this species (Fang et al., 2004). In *Araucaria angustifolia*, Transmission Electronic Microscope (TEM) analysis revealed cell wall thickening and an increase in heterochromatin in embryogenic cells throughout the cryopreservation process, which may be related to osmotic stress response and being possibly decreasing the DNA vulnerability to cleavage, therefore preserving cell integrity (Fraga et al., 2016).

### 8.3 Chromosomal and DNA variation in cryopreserved embryogenic material

During the *in vitro* culture protocol, plant cells enter in active mitosis and this genetic instability can lead to chromosomal abnormalities that can be detected in the regenerated plantlets after cryo-storage. Ploidy stability has been corroborated by flow cytometry after cryopreservation of holm oak proembryogenic masses (Martínez et al., 2022) and in somatic embryos of cork oak (Fernandes et al., 2008), as well as in the regenerated material of *Alnus glutinosa* (San José et al., 2015) and *Juglans regia* L. (Sadat-Hosseini et al., 2019). Besides, cytological analyses detected no alterations in chromosome number of *Thuja koraiensis* Nakai plantlets derived from cryopreserved somatic embryos (Ahn et al., 2019).

Regarding DNA analyses, multiloci PCR-derived markers, such as Random Amplified Polymorphic DNA (RAPD), have been used to evaluate the stability of cryopreserved embryogenic lines of several conifer species such as white spruce (*Picea glauca*) (DeVerno et al., 1999), interior spruce (*Picea glauca* x *Picea engelmannii* complex) (Cyr et al., 1994), Scots pine (*Pinus sylvestris*) (Häggman et al., 1998), *Pinus roxburghii* Sarg. (Malabadi and Nataraja, 2006), Greek fir (*Abies cephalonica* Loudon) (Aronen et al., 1999; Krajňáková et al., 2011), two hybrid firs (Salaj et al., 2010), and *Pinus nigra* (Salaj et al., 2011), as well as in plantlets derived from cryopreserved somatic embryos of *Quercus robur* (Sánchez et al., 2008). Somaclonal variation was detected in interior spruce and in RAPD profiles of white spruce, *P. roxburghii*, and Greek fir cryopreserved plant material, although DeVerno et al. (1999) reported that altered profiles were not observed in embryogenic cells until several months of culture, and not in the corresponding white spruce regenerated trees. However, altered RAPD patterns were detected in trees regenerated from somatic embryos that matured or germinated abnormally *in vitro*.

Other type of molecular marker, Inter Simple Sequence Repeats (ISSR), were applied to assess genetic fidelity of *Phoenix dactylifera* embryogenic cells (Alansi et al., 2017) and in *Thuja koraiensis* plantlets derived from cryopreserved embryogenic tissues (Ahn et al., 2019). Both multiloci markers (RAPD and ISSR) were used to analyze genetic fidelity after cryopreservation of embryogenic cells of *Platycladus orientalis* (L.) Franco (Ahn and Choi, 2017), and no variation was observed in the obtained patterns. Otherwise, no variation was observed when Simple Sequence Repeats (SSR) markers were analysed in Norway spruce embryos derived from cryopreserved embryogenic tissues (Hazubska-Przybył et al., 2013; Varis et al., 2017), however, variations were observed in SSR profiles before cryopreservation (Hazubska-Przybyl and Dering, 2017). Also, walnut ISSR profiles did not show any genetic difference among plantlets regenerated from SEs or from their parental counterpart (Sadat-Hosseini et al., 2019). In *Elaeis guineensis*, SSR profiles were identical between the regenerants and its original callus, whereas in zygotic embryos of the species, most PCR products showed polymorphism between rooted seedlings and fresh zygotic embryos before cryopreservation (Wei et al., 2023). Moreover, Fernandes et al. (2008) did not detect significant differences among control and cryopreserved *Quercus suber* somatic embryos when analyzed both Amplified Fragment Length Polymorphism and SSR patterns. Similar results were observed in two grapevine cutivars (Gribaudo et al., 2009). In contrast, SSR markers detected somaclonal variation after *Picea abies* and *Picea omorika* (Pančić) Purk. cryopreservation, although most of the observed changes were already present in the induced somatic embryos (Hazubska-Przybyl and Dering, 2017). Somaclonal variation in cryopreserved somatic embryos-derived cocoa plantlets was also detected by SSR markers on primary somatic embryos after cryostorage (Fang et al., 2009) while cocoa plantlets derived from secondary embryogenesis showed no variation in the analysed SSR patterns.

### 8.4 Epigenetic variation in cryopreserved embryogenic material

The above-described *in vitro* culture procedures which are needed for initiating the cryopreservation protocol (preculture of explants, dehydration and/or osmo- and cryoprotection), and for the after-storage recovery of embryogenic tissues, can also induce, as mentioned, epigenetic variations in plant cells. These epigenetic changes are not always deleterious, since cryopreservation has been shown to retain or even promote the regenerative capacity of embryogenic tissues (Wang et al., 2021). Epigenetic changes are mainly explained by variations in DNA methylation, in histones and in chromatin structure.

DNA methylation consists in the addition of a methyl group to the cytosine bases of DNA to form 5-methylcytosine (Tirnaz and Batley, 2019). This conserved epigenetic mark participates in shaping the genome structure, which modulates gene expression by inhibiting proteins binding to DNA, since methylation changes the conformation of the chromatin (Niederhuth and Schmitz, 2017). In plants, cytosine-methylation can occur in any context but CG is the most commonly methylated dinucleotide (Osabe et al., 2014). Methylation of specific cytosine nucleotides has been correlated with the frequency of point mutations at these sites (Mendoza-Poudereux et al., 2022), and epigenetic changes have also been considered precursors of somaclonal variation (Ibáñez et al., 2019).

DNA methylation can be measured by several approaches. Some technologies use proteins that selectively bind methylated cytosines, while others rely in amplifying DNA after digestion by methylation-sensitive restriction enzymes (methylation-sensitive amplified polymorphism, MSAP). This technology is the most frequently used for assessing epigenetic stability of cryopreserved plant material (Wang et al., 2021). Next-generation sequencing-based methods are also applied, after conversion of unmethylated cytosines to uracils using sodium bisulfite (whole-genome bisulfite sequencing, WGBS). Choosing the most appropriate methodology depends mainly on the aim of the study: assessment of the global methylation status or to find differentially methylated regions, but also depends on the quantity and purity of the available DNA (Kurdyukov and Bullock, 2016).

In this context, the cryopreservation process increased levels of global DNA methylation in *Pinus pinaster* embryogenic lines, but the initial status was recovered after thawing. This contrasted with the increased levels observed in routinely subcultured embryogenic material (Mendoza-Poudereux et al., 2022). Global methylation variation was also detected in cryopreserved *Picea glauca* embryogenic tissue (Cui et al., 2021; Gao et al., 2022), that was associated to increased embryogenic capability of recovered material. Cryopreserved cell suspensions of citrus were found to remain stable regarding ploidy constitution and DNA sequence as detected by RAPDs, while significant change in DNA methylation status was determined by MSAP assay (Hao et al., 2002). *In vitro* culture-induced variations in DNA methylation were also found in regenerates from peach palm (Heringer et al., 2013), in mahogany (*Swietenia macrophylla* King) (Harding et al., 2000), and in cocoa somatic embryos, although in this species the variability was partially reversible (Adu-Gyamfi et al., 2016).

## 9 Cryobanks of embryogenic material

Large-scale implementation of the different experimental procedures described in the present review to create cryobanks using somatic embryos are limited, and only a few examples can be given. The Spanish company TRAGSA maintained 55 embryogenic lines derived from cork oak genotypes selected for cork production (Vidal et al., 2010) by applying the procedure defined by Valladares et al. (2004). The same protocol has also been used by the MBG group to cryopreserve 124 embryogenic lines induced from cork oak genotypes identified for their tolerance to *Phytophthora cinnamomi* Rands. The aim of this cryobank is to store these valuable embryogenic lines in LN, so that the tolerance of the lines can later be confirmed in field trials. In oil palm embryogenic cultures of around 80 genotypes were cryopreserved and stored at Institute of Research for Development (IRD) in France (Dumet, 1994). Recently, Beulé et al. (2018) evaluated the embryo regeneration of rewarmed embryogenic cultures of 29 genotypes cryopreserved during 20 years at IRD Montpellier. Out of the 29 cryopreserved genotypes, 25 proliferated, showing an average of 34% embryo recovery but no decrease in recovery rate was observed compared to frequency that was reported when these genotypes were stored in LN for only 1 h. In coffee and cocoa, cryopreservation is routinely used for conserving all the new embryogenic lines generated by the Biotechnology Laboratory of the Nestlé Company (Florin et al., 1999; Engelmann, 2004), but there is no published data on the number of embryogenic lines or recovery percentages. Regarding conifers, the embryogenic cell lines are generally stored in LN at embryonal mass state (proliferation stage of the cultures); some examples of cryobanks were given by Cyr in 1999 (Cyr, 1999), and nowadays some are still maintained by research institutes and companies, mainly for *Picea* and *Pinus* species. An interesting alternative to the conservation in LN has been proposed by NEIKER group which maintained a collection of over one hundred cells lines of *Pinus radiata* at -80°C (Montalbán and Moncaleán, 2017). However, attempts to replicate it in *Picea abies* have been unsuccessful (Varis et al., 2022). As mentioned previously, SE in conifers is not feasible from adult tissues, so cryobanks are still necessary for the deployment of multivarietal forestry, to maintain the cell lines while the somatic trees are evaluated in the field (Corredoira et al., 2014).

## 10 Concluding remarks: challenges and bottlenecks

Cryopreservation is increasingly being used to store for the long-term embryogenic material of woody species as long as embryogenic cultures are available. Cryopreservation of embryogenic material is an extremely valuable tool as somatic embryos are easily recovered for future biotechnological manipulations, as well as for the preservation of biotechnological products, such as edited lines, transgenic lines or lines induced from selected trees. To date, cryopreservation protocols for more than 50 different woody species have been published. However, for each different species the protocol must be adapted by defining the best type of explant and the cryoprotective treatment. In our experience, the developmental stage of the somatic embryos is a crucial aspect in the embryo recovery frequencies obtained after LN storage (**Figure 1**) (Corredoira et al., 2014). Embryogenic cell cultures/PEMs in conifers, clumps of somatic embryos at early developmental stages in fruit and deciduous species or polyembryoids in palms are the most suitable explants for cryopreservation. Early embryogenic stages are much more resistant to storage in LN than more differentiated stages such as somatic embryos at cotyledonary stage. This is probably because cells in cotyledonary embryos display higher levels of vacuolization and differentiation than actively dividing cells present in the upper layers of PEMs or globular embryos (Sánchez-Romero et al., 2009; Corredoira et al., 2014; Popova et al., 2023), which will hamper subsequent regeneration. Regarding the cryopreservation procedure, the classic slow-cooling technique after cryoprotection with DMSO and other cryoprotectants such as sorbitol or sucrose, is the most commonly used procedure in conifers. By contrast, in other species, the greatest success has been achieved by applying vitrification-based procedures, particularly vitrification or droplet-vitrification usually both with PVS2 and following rapid immersion in LN.

Genetic stability of the cryopreserved material was initially tested by PCR-derived markers that did not detect variability in most of the species investigated. However, some epigenetic changes have been detected when total DNA methylation analyses have been performed. Nonetheless, these results contrast with the large epigenetic changes found in micropropagated embryogenic materials after prolonged subcultures, indicating the genetic stability provided by cryopreservation.

The main bottlenecks in the cryopreservation of somatic embryos are: (1) specifically for fruit and deciduous species the development of protocols for embryos at more advanced developmental stages, i.e. cotyledonary embryos (which have the advantage of being easier to manipulate) and (2) scaling up current procedures to conserve larger number of embryogenic lines. Inter-laboratory validation of protocols is necessary because, with the exception of some conifers, cork oak and oil palm, many of these procedures have been applied to only a few genotypes. Although cryopreservation procedures appear to be well defined for embryogenic cultures of several important woody species, the practical application in the creation of cryobanks is still reduced (Corredoira et al., 2014). In the coming years, efforts should be made in this way for the long-term conservation of woody plant biodiversity. In this regard, the development of new cryopreservation procedures with reduced use of cryoprotectants would facilitate the large-scale implementation of the cryopreservation of embryogenic material. Finally, data from field performance of cryogenically stored material must be made available to allow the evaluation of agronomic or forest traits in long-living tree species.

## Author contributions

DB: Conceptualization, Writing – original draft, Writing – review & editing. MM: Writing – review & editing. CS-R: Writing – original draft. IM: Writing – original draft. ES: Writing – review & editing. PM: Funding acquisition, Writing – review & editing. IA: Funding acquisition, Writing – original draft. EC: Conceptualization, Funding acquisition, Supervision, Writing – original draft, Writing – review & editing.
