# Supplementary material for: Current status of the cryopreservation of embryogenic material of woody species
Source: Front Plant Sci. 2024 Jan 17;14:1337152. doi: 10.3389/fpls.2023.1337152 (PMC10828030; doi:10.3389/fpls.2023.1337152)
Supplement: Supplementary file 1 [file Table_1.docx]

| Supplementary Material 1. Cryopreservation of embryogenic tissue by slow-cooling method in some conifer species mentioned in this review. | | | | | | |
| --- | --- | --- | --- | --- | --- | --- |
| **Species** | **Preconditioning**  **treatment** | **Cryoprotection**  **treatment** | **Slow Cooling**  **treatment** | **Recovery (%) (Number of ECLs tested)** | **Thawing**  **treatment** | **Reference** |
| *Abies alba* | SCM + 0.5 M Sorb | LCM + 0.25 M Sorb + 5% DMSO | Mr Frosty® (to -40ºC) and then, LN | 100% (4 ECLs) | 3 min at 40ºC, tissue onto filter paper and then to PM | Salaj et al. 2022 |
| *Araucaria angustifolia* | LCM + 1 M Man (4 h) | LCM + 1 M Glyc + 2 M Suc +1 M DMSO + 1% proline | Mr Frosty® (-80ºC, 4 h) and then, LN | 100 (2 ECLs) | 2 min at 40ºC, tissue centrifuged and onto filter paper and to PM | Fraga et al. 2016 |
| *Cupressus sempervirens* | SCM + 0.2 M Suc (24 h) and SCM + 0.4 M Suc (24 h) | LCM + 0.5 M Suc + 7.5% DMSO | Mr Frosty® (to -40ºC) and then, LN | 100 (1 ECL) | Until totally melted at 40ºC tissue onto filter paper and to PM (transfers 3 days and every 2 weeks) | Lambardi et al. 2018 |
| *Cryptomeria japonica* | SCM | LCM + 2M Glyc + 0.4 M Suc + 1gL^-1^ proline | Expanded polystyrene tube container (-30ºC, 6 h) | 96% (28 ECLs) | 2 min at 40ºC tissue onto filter paper and to IM (transfer 24h) | Taniguchi et al. 2020 |
| *Platycladus orientalis* | LCM + 0.4 M Sorb (24 h) | LCM + 0.4 M Sorb + 10% DMSO | Mr Frosty® (-70ºC, 24 h) and then, LN | 100% (5 ECLs) | 2-3 min at 39ºC, tissue onto nylon mesh and to IM (transfers 3, 24 h and 7 days) | Ahn and Choi 2017 |
| *Picea abies* | SCM + 0.2 M Suc (24 h) | LCM + 0.4 M Suc + 1:1 (10% PEG 6000 + 10% Glu + 10 % DMSO) | Programmable freezer (to −38°C; -0.17°C/min) and then, LN | 90% (11 ECLs) | 2 min at 37ºC tissue onto filter paper and then to PM 1M sucrose or PM (transfers 24h and every 2 weeks) | Varis et al. 2022 |
| *Picea sitchensis* | SCM + 0.4 M Sorb (48 h) | LCM + 0.4 M Sorb + 5% DMSO | Programmable freezer (to −50°C; -0.5°C/min) and then, LN | 76% (25 ECLs) | Until totally melted at 40ºC tissue onto filter paper and to PM (transfers 1, 7 days and every 2 weeks). | Gale et al. 2007 |
| *Pinus elliottii x P. caribaea* | LCM + 0.2 M Suc (24h) and LCM + 0.4 M Suc (24h) | LCM + 0.4 M Suc + 5% PEG 4000 + 5% Suc + 5% DMSO | Cell freezer container (Coolcell, Biocision) (-80ºC, 24 h) | 89% (9 ECLs) | Until totally melted at 45ºC tissue onto filter paper and then to PM (transfers every 2 weeks). | Nunes et al. 2017 |
| *Pinus nigra* | LCM + 0.5 M Suc (1 h) | LCM + 0.3 M Suc + 7.5% DMSO | Mr Frosty® (to -40ºC) and then, LN | 70% (20 ECLs) | 3-4 min at 40ºC tissue onto filter paper and then to PM | Salaj et al. 2012 |
| *Picea pungens* | CM + 0.4 M Sorb (24 h) | LCM + 0.4 M Sorb + 5% DMSO | Nalgene® cooling box (-80ºC, 2 h) | 100% (1 ECL) | 2 min at 37ºC tissue onto filter paper and to PM (transfer 24h) | Cao et al. 2022 |
| *Pinus radiata* | LCM + 0.4 Sorb (24 h) | LCM + 0.4 M Sorb + 10% DMSO | Mr Frosty® (-80ºC, 90 min) and then, LN | 100% (60 ECLs) | 2 min at 40º-45ºC, tissue onto nylon mesh and to IM with activated charcoal 1h, then to IM 24h and then to IM on nurse tissue. | Hargreaves et al. 2002 |
| *Pinus sylvestris* | SCM + 0.2 M Suc (24 h) and SCM + 0.4 M Suc (24 h) | LCM + 0.4 M Suc + 1:1 or 1:2.5 (10% PEG 6000 + 10% glu + 10 % DMSO) | Programmable freezer (to −38°C; -10°C/h) and then, LN | 92 % (96 ECLs) | 1-3 min at 37ºC, tissue washed with LM and onto filter paper to PM with 0.4, 0.2, and 0.09 M sucrose (24 h each) and then to PM every 2 weeks | Latutrie and Aronen 2013 |
| *Pinus pinaster* | LCM + 0.2 M Mal (24h) and LCM + 0.4 M Mal (24h) | LCM + 0.4 M Mal + 5% DMSO | Mr Frosty® (-80ºC, 24 h) and then, LN | 100% (3 ECLs) | Until totally melted at 45ºC tissue onto filter paper and then to PM (transfers 1 h, 24h and every two weeks) | Marum et al. 2004 |
| *Thuja koraiensis* | LCM + 0.4 M Sorb (24 h) | LCM + 0.4 M Sorb + 15% DMSO + 5% Glyc | Mr Frosty® (-70ºC, 24 h) and then, LN | 100% (5 ECLs) | As in Ahn & Choi 2017 | Ahn et al. 2019 |
| *Torreya taxifolia* | LCM + 0.4 M Sorb (24 h) | LCM + 0.4 M Sorb + 5% DMSO | Programmable freezer (to −35°C; -0.33°C/min) and then, LN | 59% (3 ECLs) | 1-2 min at 37ºC, tissue onto nylon mesh and to IM (transfers 1 and 18h) | Ma et al. 2012 |
| *Tsuga ulleungensis* | SCM + 0.09 M Suc (24 h) and SCM + 0.18 M Suc (24 h) and LCM +0.4 M Sorb (24h) | LCM + 0.4 M Sorb + 10% DMSO | Coolcell® freezing container (-70ºC, 24 h) and then LN | 100% (5 ECLs) | 2-3 min at 39ºC, tissue onto nylon mesh and to IM (transfers every 2 weeks) | Ahn et al. 2018 |
| Abbreviations: DMSO: Dimethylsulphoxide; ECLs: Established cell lines; Glu: Glucose; Glyc: Glycerol; IM: Initiation medium; LCM: Liquid culture medium; LN: Liquid nitrogen; Mal: Maltose; Man: Mannitol; PEG: Polyethyleneglycol; PM: Proliferation medium SCM: Semisolid culture medium; Sorb: Sorbitol; Suc: Sucrose. | | | | | | |
