# Supplementary material for: Current status of the cryopreservation of embryogenic material of woody species
Source: Front Plant Sci. 2024 Jan 17;14:1337152. doi: 10.3389/fpls.2023.1337152 (PMC10828030; doi:10.3389/fpls.2023.1337152)
Supplement: Supplementary file 2 [file Table_2.docx]

| **Supplementary Material 2.** Cryopreservation of embryogenic cultures in fruit tree species. In each report, information included is related to the experimental conditions giving rise to the best cryopreservation results. | | | | | | | |  |
| --- | --- | --- | --- | --- | --- | --- | --- | --- |
| **Species** | **Explant type** | **Preconditioning treatment** | **Method/Cryoprotection treatment** | **Cooling treatment** | **Thawing** | **Culture response (%)^1^** | **Reference** |  |
| *Citrus sinensis* | Cell suspensions | - | 5% DMSO + 1.2 M Suc at 0ºC for 60 min | Slow cooling at 0.5°C min^–1^ to –40°C prior to immersion in LN | Water bath at 40ºC | 70% V | Kobayashi et al. 1990 |  |
| *Citrus deliciosa* | Cell suspensions | - | 5% DMSO + 0.15 M Suc at 0ºC for 60 min | Slow cooling at 0.5°C min^–1^ to –40°C prior to immersion in LN | Water bath 40ºC | 50-60% S | Aguilar et al. 1993 |  |
| *Citrus sinensis* | SE | - | 10% DMSO for 10 min at RT (addition of DMSO lasted over a period of 1 h until the final concentration) | Slow cooling by immersion in a methanol cooling bath at RT (estimated rate of 0.5°C min^–1^) to –42°C prior to immersion in LN | Water bath at 37ºC | 30.5% S | Marín et al. 1993 |  |
| *Citrus deliciosa* | Cell suspensions | - | 5% DMSO + 0.15 M Suc at 0ºC for 60 min | Slow cooling at 0.5°C min^–1^ to –40°C prior to immersion in LN | Water bath at 40ºC | Not reported | Engelmann et al. 1994 |  |
| *Citrus* spp. | Embryogenic callus | - | 10-15% DMSO + 0.15 M Suc at 0ºC for 60 min | Slow cooling at 0.5°C min^–1^ to –40°C prior to immersion in LN | Rapid thawing | Not reported | Engelmann et al. 1994 |  |
| *Citrus* spp. | Embryogenic callus | - | 10% DMSO at 4ºC for 30 min | Slow cooling at 0.5°C min^–1^ down to –40°C followed by further cooling at 20°C min^–1^ down to –150°C, prior to immersion in LN | Water bath at 37ºC for 5 min | 100% V | Pérez et al. 1997 |  |
| *Citrus* spp. | Embryogenic callus | - | 10% DMSO at 4ºC for 30 min | Slow cooling at 0.5°C min^–1^ down to –40°C followed by further cooling at 20°C min^–1^ down to –150°C, prior to immersion in LN | Water bath at 37ºC for 5 min | Not reported | Pérez et al. 1999 |  |
| *Citrus sinensis* | Embryogenic callus | - | 10% DMSO at 4ºC for 30 min | Slow cooling at 0.5°C min^–1^ down to –40°C followed by further cooling at 20°C min^–1^ down to –150°C, prior to immersion in LN | Water bath at 37ºC for 5 min | Not reported | Olivares-Fuster et al. 2000 |  |
| *Citrus nobilis* x *C. deliciosa* | Encapsulated and non-encapsulated somatic embryos | - | 10% DMSO + 0.4 M Suc at 4ºC for 30 min and then at 0ºC for 24 h | Slow cooling in an ultra-cooling bath to -20ºC prior to immersion in LN. | Water bath at 40ºC | 51-58% S | Singh et al. 2007 |  |
| *Citrus sinensis* | Cell cultures | - | **Method:** Vitrification  **Treatment:** 60% PVS2 at 25ºC for 5 min followed by PVS2 at 0ºC for 3 min | Direct immersion in LN | Water bath at 25ºC | 83.5% S | Sakai et al. 1990 |  |
| *Citrus* spp. | Cell cultures | - | **Method:** Vitrification  **Treatment:** PVS2 at 25ºC for 3 min | Direct immersion in LN | Water bath at 25ºC | 85-98% S | Sakai et al. 1991 |  |
| *Citrus* spp. | Cell suspension | - | **Method:** Vitrification  **Treatment:** PVS2 for 3 min at RT (ca. 25ºC) | Direct immersion in LN | Water bath at 25ºC | >90% V | Hao et al. 2002 |  |
| *Citrus* spp. | SE | Encapsulation: Alginate-coated SE 1-day pregrowth in 0.75 M Suc | **Method:** Encapsulation-Dehydration  **Treatment:** Desiccation down to 20-25% moisture content | Direct immersion in LN | RT | 76-100% S | González-Arnao et al. 2003 |  |
| *Citrus sinensis* | Embryogenic calli | - | **Method:** A modified aluminum cryoplate technique  **Treatment:** PVS2 for 30 min at 0ºC | Direct immersion in LN | By immersion in unloading solution for 20 min at RT (27 ± 2ºC) | 88.7% S | Souza et al. 2017 |  |
|  |  |  |  |  |  |  |  |  |
| *Coffea arabica* | SE | Preculture in 0.5 M Suc for 18 h | 5% (v/v) DMSO + 0.5 M sucrose | Slow cooling: slow cooling at 0.5°C min^–1^ down to –40°C prior to immersion in LN | Water bath at 40ºC | 57% R | Dereuddre et al. 1994 |  |
| *Coffea canephora* | SE | Preculture in 0.3 M Suc for 18 h | 5% (v/v) DMSO + 0.3 M Suc | Slow cooling: slow cooling at 0.5°C min^–1^ down to –40°C prior to immersion in LN | Water bath at 40ºC | 28% S | Dereuddre et al. 1994 |  |
| *Coffea canephora* | SE | Preculture in 0.15 M and 1 µM ABA for 4 weeks, followed by incubation in culture medium with 0.44 M Suc and 1 µM ABA for 2 w | **Method:** Desiccation  **Treatment:** Desiccation at 75% relative humidity and 24ºC for 7 days | Direct immersion in LN | Water bath at 40ºC for 2-3 min | 80-90% V | Tessereau et al. 1994 |  |
| *Coffea arabica* | SE | - | **Method:** Vitrification  **Treatment:** Incubation in a 5% (v/v) Glyc solution containing 5% suc for 15 min, followed by a 10% (v/v) Glyc solution containing 10% Suc for a further 15 min  Dehydration in a laminar flow cabinet for 1 h | Direct immersion in LN | By immersion in liquid medium at 37ºC for 2-3 min | 66-72% S | Mycock et al. 1995 |  |
| *Olea europaea* | SE | 1 day at 30ºC in the dark before encapsulation SE | **Method:** Encapsulation-vitrification  **Treatment:** Dehydration in PVS2 for 3 h at 0ºC | Direct immersion in LN | Water bath 38ºC for 2 min | 58% G | Shibli and Al-Juboory 2000 |  |
| *Olea europaea* | SE | Preculture at 4ºC in the dark for 4 days | **Method:** Vitrification  **Treatment:** LS for 30 min at 25ºC followed by PVS2 for 90 min at 0ºC | Direct immersion in LN | 10 seconds at RT followed by 50 seconds at 40ºC in a water bath | 38% RG | Lambardi et al. 2002 |  |
| *Olea europaea* | Embryogenic callus | - | **Method:** Droplet- vitrification  **Treatment:** LS for 20 min at RT followed by 60 min incubation in PVS2 at 0º | Direct immersion in LN | By immersion in unloading solution for 15 min at RT | 100% R | Sánchez-Romero et al. 2009 |  |
| *Olea europaea* | SE clumps | 0.75 M Suc for 3 days | **Method:** Vitrification  **Treatment:** Incubation on ice for 1 h in a cryoprotectant solution (0.5 M DMSO, 1 M Suc, 0.5 M Glyc and 0.009 M proline) | Equilibration hold at 0 ◦C for 10 min, a cooling rate of 0.5 ◦C/min to −35 ◦C and a hold at−35 ◦C for 35 min, before plunging into LN | Water bath at 40ºC for 2 min | 34.6% RG | Lynch et al. 2011 |  |
| *Olea europaea* | SE | - | **Method:** Droplet-vitrification  **Treatment:** LS for 20 min at RT followed by PVS2 for 30 min at 0º | Direct immersion in LN | By immersion in unloading solution for 15 min at RT | 60% R | Bradaï et al. 2017 |  |
| *Olea europaea* | SE | 0.2 M Suc in liquid medium for 28 days | **Method:** Droplet- vitrification  **Treatment:** LS for 20 min at RT followed by PVS2 for 30 min at 0º | Direct immersion in LN | By immersion in unloading solution for 15 min at RT | 90% R | Bradaï et al. 2023 |  |
| *Persea americana* | Embryogenic cultures | - | **Method:** Droplet- vitrification  **Treatment:** LS for 20 min at RT followed by PVS2 for 60 min at 0º | Direct immersion in LN | By immersion in unloading solution for 15 min at RT | 78-100% R | Guzmán-García el al. 2013 |  |
| *Persea americana* | SE | - | **Metthod:** Droplet-vitrification  **Treatment:** LS for 20 min at RT followed by PVS2 for 60 min at 0ºC | Direct immersion in LN | By immersion in unloading solution at 37ºC | 85% V | O´Brien et al. 2016a |  |
| *Persea americana* | SE | - | **Method:** Droplet- vitrification  **Treatment:** LS with variable Suc concentration for different times at RT followed by PVS2 for 60 min at 0ºC | Direct immersion in LN | By immersion in unloading solution at 37ºC | 85-100% V | O´Brien et al. 2016b |  |
| *Theobroma cacao* | SE | Preculture on media containing 0.5 M Suc for 5 days | **Method:** Vitrification  **Treatment:** LS for 20 min at RT followed by PVS2 for 60 min at 0ºC | Direct immersion in LN | Water bath at 42ºC for 3 min | 42.9-74.5% S | Adu-Gyamfi and Wetten 2020 |  |
| *Theobroma cacao* | SE | Preculture in 0.5 M Suc medium for 5 days | **Method:** Vitrification  **Treatment:** LS for 20 min at RT followed by PVS2 for 60 min at 0ºC | Direct immersion in LN | Water bath at 42ºC for 3 min | 74.5% S | Adu-Gyamfi and Wetten 2012 |  |
| *Theobroma cacao* | SE | Preculture in 0.75-1.0 M Suc medium for 7 days | **Method:** Encapsulation-dehydration  **Treatment:** Desiccation 4 h silica exposure (16% moisture content in bead) | Direct immersion in LN | Water bath at 35ºC for 5 min | 25-72% R | Fang et al. 2004 |  |
| *Vitis vinifera* | Embryogenic cell suspensions | Liquid medium with 1 M Suc for 4 days | **Method:** Encapsulation-Dehydratation  **Treatment:** Desiccation for 2-4 h in the flow of a laminar-flow bench | Direct immersion in LN | Water bath at 40ºC for 2 min | 100% RG | González-Benito et al. 2009 |  |
|  |  |  |  |  |  |  |  |  |

**^1^**Culture response defined as viability (V), growth (G), regrowth (RG), only survival (S) or embryo recovery (R); ABA: Abscisic acid; DMSO: Dimethylsulphoxide; Glyc: Glyccerol; LN: Liquid nitrogen; LS: Loading solution; PVS2: Plant vitrification solution 2; RT: Room temperature; Suc: Sucrose; -: Not applied.
