# Supplementary material for: Current status of the cryopreservation of embryogenic material of woody species
Source: Front Plant Sci. 2024 Jan 17;14:1337152. doi: 10.3389/fpls.2023.1337152 (PMC10828030; doi:10.3389/fpls.2023.1337152)
Supplement: Supplementary file 3 [file Table_3.docx]

| **Suplementary Material 3.** Cryopreservation of embryogenic cultures in deciduous species. In each report, information included is related to the experimental conditions giving rise to the best cryopreservation results *(modified from Corredoira et al., 2017a)*. | | | | | | | |
| --- | --- | --- | --- | --- | --- | --- | --- |
| **Species** | **Explant type** | **Preconditioning treatment** | **Method/Cryoprotection**  **treatment** | **Cooling**  **treatment** | **Thawing treatment** | **Culture response^1^ (%)** | **Reference** |
| *Alnus glutinosa* | Groups of SE at globular-heart stage | 0.3 M Suc (3d) | **Method:** Vitrification  **Treatment:** Loading solution: 2 M Glyc + 0.4 M suc at RT 20 min; PVS2 at 0ºC 60 min | Direct immersion in LN | Water bath at 42ºC for 2 min | 90 R | San José et al. 2015 |
| *Aesculus hippocastanum* | SE at globular stage | 0.75 μM ABA (4d) | **Method:** Vitrification  **Treatment:** 0.5 DMSO + 0.5 M Glyc + 1 M Suc at 0ºC 60 min | Slow cooling -35ºC at 1ºC (30 min)→LN-196ºC | Water bath at 40ºC | 43 R | Jekkel et al. 1998 |
| *Aesculus hippocastanum* | SE at globular stage | 0.75 μM ABA (4d) | **Method:** Desiccation  **Treatment:** Dehydration in the laminar airflow cabinet (4h; 13% WC) | Direct immersion in LN | Water bath at 40ºC | 46 R | Jekkel et al. 1998 |
| *Aesculus hippocastanum* | SE at torpedo stage | Cold store 4ºC (5d) | **Method:** Vitrification  **Treatment:** Loading solution: 2 M Glyc + 0.4 M Suc at RT 30 min; PVS2 at 0ºC 90 min | Direct immersion in LN | Water bath at 45ºC | 70 R | Lambardi et al. 2005 |
| *Castanea sativa* | Groups of SE at globular- heart stage | 0.3 M Suc (3d) | **Method:** Vitrification  **Treatment:** PVS2 at 0ºC 60 min | Direct immersion in LN | Water bath at 42ºC for 2 min | 68 R | Corredoira et al. 2004 |
| *Castanea sativa* | Groups of SE at globular- heart stage | 0.3 M Suc (3d) → 0.7 M Suc (4d) | **Method:** Desiccation  **Treatment:** Dehydration in the laminar airflow cabinet (2h; 25% WC) | Direct immersion in LN | Water bath at 42ºC for 2 min | 33 R | Corredoira et al. 2004 |
| *Castanea sativa* | Groups of SE at globular- heart stage GT marker genes | 0.3 M Suc (3d) | **Method:** Vitrification  **Treatment:** PVS2 at 0ºC 60 min | Direct immersion in LN | Water bath at 42ºC for 2 min | 52-66 R | Corredoira et al. 2007 |
| *Castanea sativa* | Groups of SE at globular- heart stage GT thaumatin protein | 0.3 M Suc (3d) | **Method:** Vitrification  **Treatment:** PVS2 at 0ºC 60 min | Direct immersion in LN | Water bath at 42ºC for 2 min | 56-84 R | Corredoira et al. 2012b |
| *Fraxinus excelsior* | Embryogenic callus | - | **Method:** Vitrification  **Treatment:** 0.6 M Suc + 7.5% DMSO 60 min | Slow cooling  Mr.Frosty -40ºC | Water bath at 40ºC | 83 S | Ozudogru et al. 2010 |
| *Fraxinus mandshurica* | Embryogenic callus | Sorb 0.4M (20h) | **Method:** Vitrification  **Treatment:** 7.5% DMSO 90 min (0ºC) | Slow cooling -80ºC (2h)→LN-196ºC | -- | 57 R | Yu et al. 2022 |
| *Juglans nigra x J. regia* | SE at early globular stage | Cold hardening (4ºC 1w night) →5% DMSO + 0.5% proline | **Method:** Desiccation  **Treatment:** Suc (from 0.25 to 1M 24h)→Dehydration in the laminar airflow cabinet | Direct immersion in LN or slow cooling | Water bath at 40ºC for 1 min | 50-60 S | de Boucaud and Brison 1995 |
| *Kalopanax septemlobus* | Embryogenic callus | 0.3 → 0.5 → 0.7 M Suc for 17 → 3 → 3 h | **Method:** Droplet-vitrification  **Treatment:** Loading solution: 20% Glyc + 20% Suc; Vitrification solution: 33.3% Glyc + 13.3% DMSO + 13.3% EG + 20.1% Suc for  40 min at 0°C | Direct immersion in LN | Water bath at 37ºC | 70-75 R | Shin et al. 2012 |
| *Liriodendron*  *tulipifera* | Cell suspensions | Sorb 0.4 M (24h) | **Method:** Vitrification  **Treatment:** 5% DMSO (0ºC) | Slow cooling  Mr.Frosty -70ºC | Water bath at 40ºC for 2 min | 100 R | Vendrame et al. 2001 |
| *Liquidambar sp.* | Proembryogenic masses | Sorb 0.4 M (24h) | **Method:** Vitrification  **Treatment:** 5% DMSO (0ºC) | Slow cooling  Mr.Frosty -70ºC | Water bath at 40ºC for 2 min | 100 R | Vendrame et al. 2001 |
| *Melia azedarach* | Mature SE | - | **Method:** Encapsulation-Dehydration  **Treatment:** Dehydration in silica gel 3h (21-26 %WC) | Slow cooling with programmable freezer machine (1°C/min from 20°C to -30°C)→LN-196ºC | Water bath at 30ºC for 2 min | 36 R | Scocchi et al. 2007 |
| *Prunus avium* | Embryogenic callus | Suc (0.25 M 1d→ 0.5 M 1d→0.75 M 2d→ 1 M 3d) | **Method:** Desiccation  **Treatment:** Dehydration in the laminar airflow cabinet (20% WC) | Direct immersion in LN | Suc solution 1.2 M 40ºC | 89 R | Grenier-de March et al. 2005 |
| *Quercus ilex* | Groups of SE at globular stage | 0.3 M Suc (3d) | **Method:** Vitrification  **Treatment:** PVS2 at RT 30 min | Direct immersion in LN | Water bath at 42ºC for 2 min | 80 R^2^ | Barra-Jiménez et al. 2015 |
| *Quercus ilex* | Nodular embryogenic structures | 0.3 M Suc (3d) | **Method:** Vitrification  **Treatment**: PVS2 at RT 15 min | Direct immersion in LN | Water bath at 42ºC for 2 min | 63 R | Martínez et al. 2022 |
| *Quercus ilex* | Nodular embryogenic structures GT thaumatin protein | 0.3 M Suc (3d) | **Method:** Vitrification  **Treatment**: PVS2 at RT 15 min | Direct immersion in LN | Water bath at 42ºC for 2 min | 43-77 R | Cano et al. 2020 |
| *Quercus robur* | Groups of SE at globular-heart stage | 0.3 M Suc (3d) | **Method:** Vitrification  **Treatment:** PVS2 at RT 60 min | Direct immersion in LN | Water bath at 42ºC for 2 min | 70-92 R | Martínez et al. 2003  Sánchez et al. 2008 |
| *Quercus robur* | Groups of SE at globular-heart stage | 0.3 M Suc (3d) → 0.7 M Suc (4d) | **Method:** Desiccation  **Treatment:** Dehydration in the laminar airflow cabinet (2-3h; 24-34% WC) | Direct immersion in LN | Water bath at 42ºC for 2 min | 56 R | Martínez et al. 2003 |
| *Quercus suber* | Groups of SE at globular-heart stage | 0.3 M Suc (3d) | **Method:** Vitrification  **Treatment:** PVS2 at 0ºC 60 min | Direct immersion in LN | Water bath at 42ºC for 2 min | 88-93 R | Valladares et al. 2004 |
| *Quercus suber* | Groups of SE | Encapsulation 3% alginate + 0.1 M CaCl_2_→beads precultured in 0.7 M Suc (3d) | **Method:** Encapsulation-Dehydration  **Treatment:** Dehydration of beads in the laminar airflow cabinet (25-35% WC) | Direct immersion in LN | Water bath at 38ºC for 2 min | 90 S | Fernandes et al. 2008 |
| *Quercus suber* | Groups of SE at globular-heart stage GT thaumatin gene | 0.3 M Suc (3d) | **Method:** Vitrification  **Treatment:** PVS2 at 0ºC 60 min | Direct immersion in LN | Water bath at 42ºC for 2 min | 53-100 R | Cano et al. 2021 |
| **^1^**Culture response defined as only survival (S) or embryo recovery (R); **^2^**Embryo differentiation was only observed after 24h LN; their differentiation ability was completely lost after 4 weeks in LN. Abbreviations: ABA: Abscisic acid; DMSO: Dimethylsulphoxide; EG: Ethyleneglycol; GT: Genetic transformation; Glyc: glycerol; LN: Liquid nitrogen; PVS2: Plant vitrification solution 2; RT: Room temperature; SE: Somatic embryos; Sorb: Sorbitol; Suc: Sucrose; WC: Water content; -: Not applied; --: Not mentioned. | | | | | | | |
