# Supplementary material for: Current status of the cryopreservation of embryogenic material of woody species
Source: Front Plant Sci. 2024 Jan 17;14:1337152. doi: 10.3389/fpls.2023.1337152 (PMC10828030; doi:10.3389/fpls.2023.1337152)
Supplement: Supplementary file 4 [file Table_4.docx]

| **Suplementary Material 4.** Cryopreservation of embryogenic cultures in palm tree species. In each report, information included is related to the experimental conditions giving rise to the best cryopreservation results. | | | | | | | |
| --- | --- | --- | --- | --- | --- | --- | --- |
| **Species** | **Explant type** | **Preconditioning treatment** | **Method/Cryoprotection**  **treatment** | **Cooling**  **treatment** | **Thawing treatment** | **Culture response^1^ (%)** | **Reference** |
| *Bactris gasipaes* | Embryogenic clusters | 0.3 M Suc (1h) → 0.6 M Suc (1h) | **Method:** Droplet-vitrification  **Treatment:** PVS3 120 min | Direct immersion LN | Water bath at 40ºC for 2 min | 52 R | Heringer et al. 2013a |
| *Bactris gasipaes* | SE | 0.3 M Suc (1h) → 0.6 M Suc (1h) | **Method:** Vitrification  **Treatment:** PVS3 240 min | Direct immersion LN | Water bath at 40ºC for 2 min | 37 R | Heringer et al. 2013b |
| *Cocos nucifera* | Embryogenic callus | 0.75 M Suc (3d) | **Method:** Encapsulation-dehydration  **Treatment:** Dehydration in silica gel 20h (27-30 %WC) | Direct immersion LN | Water bath at 40 °C for 3 min | 25 R | Welewanni et al. 2020 |
| *Elaeis guineensis* | Clumps of embryoids | 0.75 M Suc (7d) | - | Slow cooling with programmable freezer machine: 0.5 °C/min to −20, −40 or −100 °C→LN | Water bath at 40 °C for 1 min | 8.3 R | Engelmann and Dereuddre 1988 |
| *Elaeis guineensis* | Clumps of embryoids | 0.75 M Suc (7d) | - | Slow cooling versus  direct immersion LN | Water bath at 40 °C for 1 min | 0-33 R  (average 12.5) | Engelmann 1990 |
| *Elaeis guineensis* | Clumps of SE | 0.75 M Suc (7d) | **Method:** Desiccation  **Treatment:** Dehydration in silica gel 16h (19-36 %WC) | Direct immersion LN | Water bath at 40ºC for 2 min | 13-53 S | Dumet et al. 1993 |
| *Elaeis guineensis* | Polyembryoids | 0.5 M Suc (12h) | **Method:** Vitrification  **Treatment:** Loading solution: 10% DMSO + 0.7M Suc; PVS2 5 min 26ºC | Direct immersion LN | Water bath at 38 °C for 1.5 min | 45 S | Suranthran et al. 2012 |
| *Elaeis guineensis* | Isolated polyembryoids^2^ | 0.5 M Suc (12h) | **Method:** Droplet-vitrification  **Treatment:** Loading solution: 10% DMSO + 0.7M Suc; PVS2 10 min | Direct immersion LN | Aluminium foils in a solution 1.2 M Suc for 15 min RT | 68 R | Gantait et al. 2015 |
| *Elaeis guineensis* | Clumps of SE | 0.75 M Suc (7d) | **Method:** Encapsulation-dehydration  **Treatment:** Dehydration in silica gel 20h (19-35 %WC) | Direct immersion LN | -- | 33 R after 20 years in LN | Beulé et al. 2018 |
| *Elaeis guineensis* | Polyembryoids | Gradual exposure to 0.3, 0.5, 0.75 and 1 M Suc for 7 days | **Method:** Encapsulation-dehydration  **Treatment:** Dehydration in a laminar airflow cabinet (9h; 23% WC) | Direct immersion LN | Water bath for 2–3 min at 40 °C | 73 R | Palanyandy et al. 2020 |
| *Phoenix dactilifera* | Proembryogenic masses | 0.5 M Suc (3d) | **Method:** Droplet-vitrification  **Treatment:** PVS2 30 min 0ºC | Direct immersion LN | Aluminium foils in a solution 1.2 M suc for 15 min RT | 63 R | Fki et al. 2011 |
| *Phoenix dactilifera* | Proembryogenic masses | DV: No preculture  VC:0.5 M Suc (3d) | **Method:** Droplet-vitrification and V cryoplate  **Treatment:**  DV→PVS2 30-60 min  VC→ desiccation 60–120 min | Direct immersion LN | DV: Aluminium foils in a solution 1.2 M Suc for 15 min  VC: Cryoplate in a solution 1.2 M suc for 15 min RT | DV: 87-99 R  VC: 67-96 R | Salma et al. 2014 |
| *Phoenix dactilifera* | Clumps of PEMs | 0.5 M Suc (3d) | **Method:** D cryoplate  **Treatment:** Loading solution: 2M Glyc + 0.4M Suc→ Dehydration in a laminar airflow cabinet (90-120 min; 40-50%WC) | Direct immersion LN | Cryoplate in a solution 1.2 M suc for 15 min RT | 75-96 R | Salma and Engelmann (2017) |
| *Phoenix dactilifera* | Proembryogenic masses | 0.5 M Suc (48h) | **Method:** Encapsulation-dehydration  **Treatment:** Dehydration in a laminar airflow cabinet (2-4; 49% WC) | Direct immersion LN | Water bath at 38 °C for 2-4 min | 53 R | Alansi et al. 2019 |
| **^1^**Culture response defined as only survival (S) or embryo recovery (R); Polyembriods: comprising of haustorium and torpedo-shaped structures. Abbreviations: DMSO: Dimethylsulphoxide; DV: Droplet-Vitrification; Glyc: Glycerol; LN: Liquid nitrogen; RT: Room temperature; PVS2: Plant vitrification solution 2; PVS3: Plant vitrification solution 3; SE: Somatic embryos; Suc: sucrose; VC: V Cryoplate; WC: Water content; -: Not applied; --: Not mentioned. | | | | | | | |
