# Supplementary material for: Current status of the cryopreservation of embryogenic material of woody species
Source: Front Plant Sci. 2024 Jan 17;14:1337152. doi: 10.3389/fpls.2023.1337152 (PMC10828030; doi:10.3389/fpls.2023.1337152)
Supplement: Supplementary file 5 [file Table_5.docx]

| **Supplementary Material 5.** Genetic stability assessment during and after cryopreservation of embryogenic lines in woody species. | | | |
| --- | --- | --- | --- |
| **Species** | **Assessment method** | **Instability detected** | **Reference** |
| *Abies alba x A. cephalonica*  *Abies alba x A. numidica* | RAPD | No | Salaj et al. 2010 |
| *Abies cephalonica* | RAPD | Yes | Aronen et al. 1999 |
|  | RAPD | Yes | Krajnakova et al. 2011 |
| *Alnus glutinosa* | Flow cytometry | No | San José et al. 2015 |
| *Araucaria angustifolia* | TEM assays | Changes in cell wall and heterochromatin | Fraga et al. 2016 |
| *Bactris gasipaes* | Global DNA methylation | Changes depending on the cryopreservation method | Heringer et al. 2013a |
| *Citrus spp* | Phenotype of regenerants  Chromosome counting  RAPD | No  No  No | Pérez et al. 1998 |
|  | MSAP | Yes | Hao et al. 2002 |
| *Elaeis guineensis* | SSR  Antioxidant enzymes | No  Increased during the cryopreservation protocol | Wei et al. 2023 |
| *Melia azedarach* | Phenotype of regenerants | No | Scocchi et al. 2007 |
| *Juglans regia* | Flow cytometry  ISSR | No  No | Sadat-Hosseini et al. 2019 |
| *Olea europaea* | Regeneration capability of embryogenic lines | No | Bradai and Sanchez-Romero 2021 |
| *Phoenix dactylifera* | ISSR | No | Alansi et al. 2017 |
| *Picea abies* | SSR | No | Hazubska-Przyby et al. 2013 |
|  | SSR | No | Varis et al. 2017 |
| *Picea abies* | SSR | Changes before cryopreservation | Hazubska-Przybył and Dering 2017 |
| *Picea glauca engelmanni complex* | SSR | Yes | Cyr et al. 1994 |
| *Picea glauca* | RNAseq  Global DNA methylation | Yes | Gao et al. 2022 |
|  | Global DNA methylation  Expression antioxidant | Yes  Yes | Cui et al. 2021 |
|  | RAPD | Changes in embryogenic lines but not in plants | De Verno et al. 1999 |
| *Picea omorika* | SSR | Yes | Hazubska-Przybyl and Dering 2017 |
| *Pinus nigra* | RAPD | No | Salaj et al. 2011 |
| *Pinus pinaster* | Global DNA methylation | Changes during cryopreservation but recovered after thawing | Mendoza-Poudereux et al. 2022 |
| *Pinus roxburghii* | RAPD | Yes | Malabadi and Nataraja 2006 |
| *Pinus sylvestris* | RAPD | No | Häggman et al. 1998 |
| *Platycladus orientalis* | RAPD  ISSR | No  No | Ahn and Choi 2017 |
| *Quercus ilex* | Flow cytometry | No | Martínez et al. 2022 |
| *Quercus robur* | RAPD | No | Sanchez et al. 2008 |
| *Quercus suber* | Flow cytometry  AFLP  SSR | No  No  No | Fernandes et al. 2008 |
| *Swietenia macrophylla* | RFLP  DNA methylation | No  Yes | Harding et al. 2000 |
| *Theobroma cacao* | Phenotype | Yes | Wetten et al. 2009 |
|  | Phenotype | No | Fang et al. 2004 |
|  | MSAP | Yes | Adu-Gyamfi et al. 2016 |
|  | SSR in secondary SE | No | Fang et al. 2009 |
| *Thuja koraiensis* | Chromosome number  ISSR | No  No | Ahn et al. 2019 |
| *Vitis vinifera* | Phenotype of plants | No | Vasanth and Vivier 2011 |
|  | Leaf phenotype | No | Wang et al. 2002 |
|  | AFLP | No | Gribaudo et al. 2009 |
|  | SSR | No |  |

Abbreviations: AFLP, Amplified Fragment Length Polymorphism; ISSR, Inter-Simple Sequence Repeats; MSAP, Methylation-sensitive Amplified Polymorphism; RAPD, Random Amplified Polymorphic DNA; RFLP, Restriction Fragment Length Polymorphism; SSR, Simple Sequence Repeats; TEM, Transmission Electron Microscope.
