# Supplementary material for: Current status of the cryopreservation of embryogenic material of woody species
Source: Front Plant Sci. 2024 Jan 17;14:1337152. doi: 10.3389/fpls.2023.1337152 (PMC10828030; doi:10.3389/fpls.2023.1337152)
Supplement: Supplementary file 6 [file DataSheet_1.pdf]

REFERENCES OF SUPPLEMENTARY MATERIAL

Alansi, S., Al-Qurainy, F., Nadeem, M., Khan, S., Tarroum, M., Alshameri, A., et al. (2019). Cryopreservation: A tool to conserve date palm in Saudi Arabia. Saudi J. Biol. Sci. 26, 1896–1902. doi: 10.1016/j.sjbs.2019.02.004

Alansi et al. 2019 is mentioned in supplementary material Table 4.

Grenier-de March, G., de Boucaud, M. T., and Chmielarz, P. (2005). Cryopreservation of *Prunus avium* L. embryogenic tissues. CryoLetters 26, 341–348.

Grenier de March et al. 2005 is mentioned in supplementary material Table 3.

Heringer, A. S., Steinmacher, D. A., Schmidt, É.C., Bouzon, Z. L., and Guerra, M. (2013b). Survival and ultrastructural features of peach palm (*Bactris gasipaes* Kunth) somatic embryos submitted to cryopreservation through vitrification. Protoplasma 250, 1185–1193. doi: 10.1007/s00709-013-0500-4

Heringer et al. 2013b is mentioned in supplementary material Table 4.

Pérez, R. M., Mas, O., Navarro, L., and Duran-Vila, N. (1999). Production and cryoconservation of embryogenic cultures of mandarin and mandarin hybrids. Plant Cell Tiss Organ Cult. 55, 71–74. doi: 10.1023/A:1026433405701

Pérez et al. 1999 is mentioned in supplementary material Table 2.

Lambardi, M., Lynch, P. T., Benelli, C., Mehra, A., and Siddika, A. (2002). Towards the cryopreservation of olive germplasm. Adv. Hortic. Sci. 16, 165–174.

Lambardi et al. 2002 are mentioned in supplementary material Table 2.

Suranthran, P., Gantait, S., Sinniah, U. R., Subramaniam, S., Alwee, S. S. R. S., and Roowi, S. H. (2012). Efect of loading and vitrifcation solutions on survival of cryopreserved oil palm polyembryoids. Plant Growth Regul. 66, 101–110. doi: 10.1007/s10725-011-9633-7

Suranthran et al. 2012 is mentioned in supplementary material Table 4.
